# Light energy partitioning, photosynthetic efficiency and biomass allocation in invasive *Prunus serotina* and native *Quercus petraea* in relation to light environment, competition and allelopathy

**DOI:** 10.1007/s10265-018-1009-x

**Published:** 2018-02-07

**Authors:** Piotr Robakowski, Ernest Bielinis, Kerrie Sendall

**Affiliations:** 1 Department of Forestry, Poznan University of Life Sciences, Wojska Polskiego 71E St., 60-625 Poznan, Poland; 2 Unit of Forestry and Forest Ecology, Department of Environmental Management and Agriculture, University of Warmia and Mazury in Olsztyn, PL Lodzki 2, 10-727 Olsztyn, Poland; 3 Department of Biology, Georgia Southern University, P.O. Box 8042, Statesboro, GA 30460 USA

**Keywords:** Acclimation to light, Chlorophyll *a* fluorescence, Competition, Energy partitioning, Net CO_2_ assimilation rate, Photosynthetic efficiency

## Abstract

This study addressed whether competition under different light environments was reflected by changes in leaf absorbed light energy partitioning, photosynthetic efficiency, relative growth rate and biomass allocation in invasive and native competitors. Additionally, a potential allelopathic effect of mulching with invasive *Prunus serotina* leaves on native *Quercus petraea* growth and photosynthesis was tested. The effect of light environment on leaf absorbed light energy partitioning and photosynthetic characteristics was more pronounced than the effects of interspecific competition and allelopathy. The quantum yield of PSII of invasive *P. serotina* increased in the presence of a competitor, indicating a higher plasticity in energy partitioning for the invasive over the native *Q. petraea*, giving it a competitive advantage. The most striking difference between the two study species was the higher crown-level net CO_2_ assimilation rates (A_crown_) of *P. serotina* compared with *Q. petraea*. At the juvenile life stage, higher relative growth rate and higher biomass allocation to foliage allowed *P. serotina* to absorb and use light energy for photosynthesis more efficiently than *Q. petraea*. Species-specific strategies of growth, biomass allocation, light energy partitioning and photosynthetic efficiency varied with the light environment and gave an advantage to the invader over its native competitor in competition for light. However, higher biomass allocation to roots in *Q. petraea* allows for greater belowground competition for water and nutrients as compared to *P. serotina*. This niche differentiation may compensate for the lower aboveground competitiveness of the native species and explain its ability to co-occur with the invasive competitor in natural forest settings.

**Electronic supplementary material:**

The online version of this article (10.1007/s10265-018-1009-x) contains supplementary material, which is available to authorized users.

## Introduction

Competition among trees results from genetically founded species-specific and ontogenetic differences in growth dynamics, maximum net CO_2_ assimilation rate (A_max_), leaf nitrogen concentration (N_mass_) and requirements for nutrients, water and light (Craine and Dybzinski 2013; Reich et al. 1992). On a global scale across biomes and plant functional groups there is evidence that key leaf traits such as specific leaf area (SLA), N_mass_, A_max_ and dark respiration (R_d_) are positively related (Reich et al. 1998). The species and individuals which grow faster and overtop competitors are better adapted to win the battle for light, thus increasing their photosynthetic and growth performance (Balandier 2005; Novoplansky 2009; Peet and Christensen 1987). In natural conditions, however, it is difficult to distinguish the effects of different environmental factors on plant performance from plant–plant competitive interactions or allelochemical relations. The outcomes of plant competition can be identified more easily in controlled conditions. Here, in a pot experiment, we simulated the relationship between *Quercus petraea* and *Prunus serotina* seedlings under the canopy of a Scots pine forest, which has been observed in natural conditions.

Growth and photosynthetic competition occur when a plant is able to increase or maintain rates of growth and/or photosynthesis, while reducing rates of its competitor (Grime 1974, 1977). A major aspect of photosynthetic competition involves competing for light, water and nutrients required for photosynthesis. An outcome of competition may be the ability of a plant to acclimatize or adapt its photosynthetic apparatus and functions to meet the altered availability of photosynthetic substrates. A species may gain an advantage over its competitor via its capacity to acclimate from low to high light regimes, its higher photosynthetic capacity in high light, or higher leaf anatomical plasticity in response to changing light (Baker 1965; Daehler 2003; Oguchi et al. 2008; Valladares et al. 2000). For example, in cool-temperate deciduous forests, a trade-off between photosynthetic plasticity and shade tolerance was observed in response to gap formation and increased light availability (Oguchi et al. 2017). Gap-dependent species were more plastic and developed leaves with new traits that allowed for fast growth, whereas gap-independent species sacrified photosynthetic plasticity in order to maintain shade tolerance (Oguchi et al. 2017). Additionally, invasive species exhibit higher plasticity in terms of biomass allocation and many leaf traits in response to nutrient availability (Funk 2008). A prior comparison of invasive *P. serotina* and native *Q. petraea* growth dynamics showed that the former is a superior competitor for light under introduced conditions (Robakowski and Bielinis 2011). Here, we address whether the competitive advantage observed in invasive *P. serotina* occurs as a result of higher photosynthetic capacity.

The leading hypothesis explaining the mechanism of plant invasion (the enemy release hypothesis) states that invasive species escape from natural enemies and develop novel competitive abilities in a new environment (Bais et al. 2003; Keane and Crawley 2002). For example, Reinhart et al. (2003) provided evidence that the invasiveness of *P. serotina* in Europe was due in part to its escape from *Pythium* spp., a primary competitor in its native range. Our observations and preliminary results (Robakowski and Bielinis 2011), however, suggest that the resource-enemy release hypothesis, in which the enemy release hypothesis and increased resource availability act in concert, may more accurately explain the invasion of *P. serotina* in Europe (Blumenthal 2005).

It is important to note that a great number of species perform similarly in introduced ranges when compared with their conspecific populations in their native range (Alba and Hufbauer 2012; Parker et al. 2013). Species that perform similarly in introduced and native habitats are generally referred to as non-native, while those that are more competitive in introduced habitats are referred to as invasive (Parker et al. 2013; van Kleunen et al. 2010). Under new environmental conditions, an invasive species may be larger in size, exhibit higher reproductive performance and resource use efficiency, and have lower biomass production costs than in its native habitat. Invasive plants often inhibit the growth of competitors and are highly efficient at capturing and utilising light, water, mineral nutrients and space (Heberling and Fridley 2013; van Kleunen et al. 2010).

Allelochemical activity has been proposed as an alternative explanation for the success of some invasive species (Callaway and Aschehoug 2000; Hierro and Callaway 2003). In an introduced environment, an invasive species may use a “new weapon” to compete more efficiently with native species (Yuan et al. 2013). For example, some invasive species use allelochemicals volatilized from leaves or released from roots as exudate in soil (Koutika et al. 2007; Ubalua 2010). Bais et al. (2003) showed that invasive *Centaurea maculosa* inhibited growth and germination of native species in field soils with the phytotoxin (−)-catechin released from its roots. In the present study, we investigate the effect of a cyanogenic glycoside, prunasin, which is produced by the invasive *P. serotina* and is known for its allelochemical properties (Leavesley et al. 2008; Robakowski et al. 2016; Swain et al. 1992; Vetter 2000). Our earlier paper (Robakowski et al. 2016) was focused on seasonal changes in root prunasin concentrations of *P. serotina* and correlations among prunasin concentration and different ecophysiological parameters.

To understand the mechanism of invasion, invasive species have been compared with their native congeners with respect to growth and photosynthetic traits (McDowell 2002; van Kleunen et al. 2010). The advantage of comparing congeners rather than unrelated species is that the comparison of congeners provides more insight into which traits actually play a role in the invasiveness of a species and which are merely coincidental (Mack 1996; McDowell 2002). Photosynthetic and growth characteristics of invasive plants have also been compared between invasive species and non-invasive, unrelated native species (Baruch and Goldstein 1999; Pattison et al. 1998). Functionally similar species are more likely to compete (Abrams 1983), but if an invasive species and a native species are functionally similar, the invasive species may lack the competitive advantage needed to outgrow the native species (Davis et al. 2000). *P. serotina* and *Q. petraea* used in our study are broadleaved deciduous trees originating from different families, but they share the same ecological niche in European forests.

Invasive species often have different suites of leaf traits when compared with native species. For instance, invasive species have been shown to have higher photosynthetic capacity, higher photosynthetic nitrogen and water use efficiency (PNUE and WUE, respectively), greater instantaneous photosynthetic energy-use efficiency, lower respiration costs, lower construction costs of leaf tissue, and lower leaf mass to area ratio (LMA) when compared with native species (Heberling and Fridley 2013; McDowell 2002). Invasive species, however, do not have fundamentally different carbon capture strategies from natives, but are instead positioned further along the leaf economics spectrum towards faster growth strategies (Leishman et al. 2007). On a global scale, plants with lower LMA are more efficient at light interception per unit leaf dry mass than those with higher LMA (Wright et al. 2004). Species that best compete for light under shaded conditions are highly efficient at light capture due to higher allocation of resources to light harvesting complexes, increased chlorophyll concentrations and reductions of chlorophyll a/b ratios (Hikosaka and Terashima 1996; Lei and Lechowicz 1998). In high light, high leaf nitrogen concentration and photosynthetic capacity, as well as effective mechanisms of dissipating excess energy, can confer an advantage in competition for resources (Demmig-Adams and Adams 1996, 2006; Heberling and Fridley 2013).

The photosynthetic performance of our study trees was determined by comparing the fraction of leaf absorbed light energy transferred to photochemistry (Φ_PSII_) vs. heat (Φ_NPQ_) and fluorescence (Φ_f,D_). This partitioning of leaf absorbed light energy depends on factors such as temperature and light availability. Species and individuals allocating high levels of energy to photochemistry have potentially higher net CO_2_ assimilation rates compared to plants that dissipate more energy as heat and fluorescence (Genty et al. 1989; Maxwell and Johnson 2000). In the present study, we have posited that *P. serotina* and *Q. petraea* will differ in terms of proportions of leaf absorbed light energy transferred to different processes, with a higher proportion of energy allocated to Φ_PSII_ in the species with a competitive advantage (Funk 2008). However, under non-optimal conditions, as was the case in the strong shade and high light treatments in our experiment, photochemical energy can be used in several alternative pathways such as photorespiration, the Mehler reaction or the xanthophyll cycle, which may lead to a reduction in the rate of net CO_2_ assimilation (Demmig-Adams et al. 1996; Demmig-Adams and Adams 2006; Demmig-Adams and Niyogi 1999). In addition to variation in leaf absorbed light energy partitioning, we expected our different competition and allelochemical treatment combinations (see details below) to influence leaf structure (LMA), physiology (respiration, net CO_2_ assimilation rates), and biomass allocation to different organs as was shown for *Picea abies* and *Fagus sylvatica* in Kozovits et al. (2005).

The aim of our study was to determine the mechanisms of competitive interactions between invasive *P. serotina* and native *Q. petraea*. To achieve this, we measured leaf absorbed light energy partitioning and photosynthetic and growth rates of seedlings in response to variations in light environment, competition, and allelopathic effects by mulching with *P. serotina* leaves. The following hypotheses were tested: (1) invasive *P. serotina* will invest more energy in Φ_PSII_ rather than in Φ_NPQ_ and Φ_f,D_ when growing in competition with native *Q. petraea* than it will when growing in monoculture. In contrast, native *Q. petraea* will not increase energy transfer to Φ_PSII_ when growing in the presence of *P. serotina* and/or allelopathic compounds. Lower losses of leaf absorbed light energy and higher Φ_PSII_ will give an advantage to the invasive over the native species in photosynthetic performance and growth. This hypothesis is supported by the results of Funk (2008) who showed that effective quantum yield of fluorescence can be higher in invasive compared with native species. Alternatively, Hendrickson et al. (2004) found that partitioning of leaf absorbed light energy was driven more by light availability than by interspecific competition. (2) When competing with *Q. petraea, P. serotina* will enhance its photosynthetic capacity and resource use efficiency to increase growth at lower costs and compete for aboveground resources more effectively than the native species. In contrast, in the presence of the invasive competitor and/or allelopathic effects of *P. serotina* leaves, *Q. petraea* will decrease its photosynthetic capacity and resource use efficiency. Earlier results from other studies suggest that there is not a clear difference between native and invasive species in terms of absolute rates of photosynthetic capacity and resource use efficiency, but invasives do appear to have higher plasticity of the latter (Daehler 2003; Funk 2008). (3) Effects of competition and/or allelopathy on photosynthesis will be more pronounced under high light than under intermediate and low light levels. In high light, the competing species will assimilate more carbon and thus be able to allocate more biomass to photosynthesis and growth, which will result in intense competition for space and light. In contrast, under strong shade, seedlings will reduce growth and photosynthesis independent of species or treatment and competition will be minimal or absent. Oguchi et al. (2017) found that species-specific photosynthetic responses to increased light availability in forest trees can influence their interspecific competition. Based on 79 independent native-invasive plant comparisons, Daehler (2003) stated that increased resource availability increased the performance of invasive species over that of natives. (4) *P. serotina* leaves used as a source of allelochemicals in mulching will inhibit growth, respiration and photosynthesis of native *Q. petraea*. Leaves contain the allelochemical prunasin that, after being hydrolyzed, decomposes into hydrogen cyanide (HCN), a respiratory poison that inhibits the activity of metalloenzymes such as cytochrome *c* oxidase (Leavesley et al. 2008; Swain et al. 1992; Vetter 2000). (5) At the juvenile stage, invasive *P. serotina* will experience higher growth rates and higher biomass allocation to leaves than roots, while native *Q. petraea* will allocate more growth toward roots at the expense of leaves. This hypothesis is based on our observations of natural regeneration of both species in forest understories and preliminary research showing that the growth rates of *P. serotina* can be several times greater than that of *Q. petraea* (Robakowski and Bielinis 2011). It is also supported by earlier studies from Kozovits et al. (2005) who showed that *Picea abies* was a stronger competitor than *Fagus sylvatica* due to higher above-ground biomass increments in mixed culture than in monoculture.

## Materials and methods

### Material

*Prunus serotina* (Ehrh.) Borkh. is a deciduous tree or a small understorey shrub that is native to North America (Forestry Compendium 2005; Marquis 1990). As juveniles, the species is classified as moderately shade tolerant, showing the highest growth rate in 25% of full sun and only slightly lower in 100% (Robakowski, unpublished). The species exhibits the “sit and wait” life strategy, i.e. seedlings are able to survive beneath the dense canopy, but grow quickly in the high light of forest clearings and gaps (Closset-Kopp et al. 2011). The species is able to rapidly occupy new territories due to mass and frequent seed production, propagation of seeds by birds, resprouting from trunks and roots, and high rates of annual growth. It is an aggressive colonizer, capable of rapidly overtopping native tree species when introduced to new habitats (Csiszár et al. 2013; Halarewicz 2011; Möllerová 2005).

*Quercus petraea* (Matt.) Liebl. is an economically important deciduous broadleaved tree occurring in Europe. As juveniles, *Q. petraea* is classified as shade-tolerant. In Poland and Germany, natural regeneration of *Q. petraea* is favoured beneath the canopy of *Pinus sylvestris* (L.) stands of different age (Kenk 1993), occurring together with the natural regeneration of *P. serotina*.When naturally regenerating in forest understories such as our experimental forest, *P. serotina* tends to be at least twice as common (up to 120 seedlings per m^2^) as *Q. petraea*, and in this study we were attempting to simulate this competition. Thus, we planted a higher number of seedlings of the invasive species in each competition treatment since it accurately reflected the observed pressure of *P. serotina* on *Q. petraea*. The greater regeneration density of *P. serotina* compared with *Q. petraea* is likely due to more frequent and abundant seed production, accumulation of seeds in soil and high seed germination capacity even after 5 years of being in soil, distribution of seeds by birds, higher capacity of vegetative regeneration and much higher growth rates at the juvenile life stage. Additionally, our observations indicate that in the university experimental forest *P. serotina* is not browsed by deer and roes, while *Q. petraea* seedlings and saplings are often damaged by these animals. In addition, *Q. petraea* acorns are eaten by deer, wild boar, squirrels and other animal species. *P. serotina* seeds, leaves and roots contain the cyanogenic glycosides amygdalin and/or prunasin, which repel animals.

In October 2011, acorns of *Q. petraea* were collected in a selected seed stand located in Jarocin Forest Division, Western Poland. At the end of February 2012, acorns were potted in a peat and perlite substrate (3:1; v/v) and grown in a plastic tunnel without heating at the Jarocin forest nursery (51°58′45″N; 17°29′8″E). Seedlings were moved outdoors at the end of March. *P. serotina* seedlings originated from the natural regeneration occurring in “Zielonka” Forest, 27 km from Poznan, Western Poland (52°33′29″N; 17°06′18″E). At the beginning of May 2012, 1-year-old seedlings were dug up and transported with roots thoroughly covered with humus to Poznan University of Life Sciences Dendrological Garden.

*Quercus* and *Prunus* seedlings were potted using 225 7-l pots filled with a mixture of pH-neutral sand, peat and humus (1/1/1; v/v/v). For better drainage, a small amount of gravel was added at the bottom of pots. Before planting, 100 g of fresh *P. serotina* leaves, cut into small pieces (~ 0.25 cm^2^), were added to each of 90 pots and mixed with the substrate to enhance the expected allelopathic reaction. Mulching was repeated once a month from May to September with 10 g of freshly cut *P. serotina* leaves. Leaves of *P. serotina* decomposed almost entirely in pot within around 30 days. In leaves used for mulching, prunasin concentrations were 2.86 ± 048 and 15.9 ± 4.89 mg g^−1^ FW (mean ± SE, FW—fresh weight, *n* = 4) in May and August, respectively. The lowest leaf prunasin concentration was 1.93, and the highest value was 29.93 mg g^−1^ FW. We used five treatment combinations using different numbers of seedlings and different mulching scenarios: (1) Q: three seedlings of *Quercus petraea*; (2) P: three seedlings of *P. serotina*; (3) Q + L: three seedlings of *Q. petraea* + mulching with *P. serotina* leaves; (4) Q + P: three seedlings of *Q. petraea* + six seedlings of *P. serotina*; (5) Q + P + L: three seedlings of *Q. petraea* + six seedlings of *P. serotina* + mulching. As explained above, *P. serotina* seedlings tend to be at least twice as common as *Q. petraea* seedlings when regenerating in natural conditions. Thus, in order to ensure that adequate competitive pressure on the native species was achieved in our treatments, we used six *P. serotina* seedlings and three *Q. petraea* seedlings in both competition treatments. All combinations are schematically shown in Robakowski et al. (2016). From this point forward, we use variations on these treatment combinations to denote which species’ measurements are being presented; for example, P + Q indicates that traits presented were measured on *P. serotina* seedlings that were competing with *Q. petraea* seedlings, while Q + P indicates that traits presented were measured on *Q. petraea* seedlings that were competing *P. serotina* seedlings.

In May 2012, the seedlings were fertilized using 15 g of slow-releasing fertilizer ‘Osmocote Exact Standard’ (N, P, K, Mg—15:9:12:2 and microelements) per pot. Every 2 days, seedlings were watered to field capacity using an automatic irrigation system. Watering was less intensive in the low light regime (10% of full light) and was stopped when it was raining. Each month, a subset of pots were emptied and seedlings were harvested for biomass allocation analyses and substrate moisture content was observed. In September, water content in soil was measured gravimetrically and there were no significant differences among light treatments (*P* = 0.07). Q + L had the highest (46%) and Q + P the lowest (32%) water content in substrate (*P* < 0.001). Potted seedlings were grown from half May to the end of November.

### Experimental design

225 pots were distributed into three blocks (75 pots per block) and three light treatments, established using a shading net: LL (10% of full sun), ML (25% of full sun) and HL (100% of full sun). The spectral proprieties of the material used for the shading net have been described in Robakowski (2005). There were 25 pots in each block by light treatment. Five combinations of seedlings with or without mulching (Table 1) were distributed in split-plots (five pots per block, light treatment and combination). Each combination was repeated five times in each plot i.e. 225 experimental units (5 repetitions × 5 combinations × 3 light treatments × 3 blocks = 225 pots).

**Table 1:** Effects of light environment and treatment combinations of *Prunus serotina* and *Quercus petraea* seedlings with or without mulching on quantum yield of constitutive fluorescence and thermal dissipation (Φ_f,D_), quantum yield of thermal energy dissipation (Φ_NPQ_), and quantum yield of PSII photochemistry (Φ_PSII_) at PPF = 295 µmol m^−2^ s^−1^ (mean ± SE; *n* = 6)

Effect	Φ_f,D_	Φ_NPQ_	Φ_PSII_
*Prunus serotina* energy partitioning			
LL	0.28 ± 0.01a	0.50 ± 0.02a	0.22 ± 0.01a
ML	0.23 ± 0.01b	0.47 ± 0.03ab	0.30 ± 0.02b
HL	0.19 ± 0.01b	0.42 ± 0.03b	0.39 ± 0.03c
P	0.22 ± 0.01a	0.49 ± 0.02a	0.29 ± 0.02a
P + Q	0.24 ± 0.02a	0.49 ± 0.02a	0.27 ± 0.02a
P + Q + L	0.24 ± 0.01a	0.41 ± 0.03b	0.35 ± 0.03b
*Quercus petraea* energy partitioning			
LL	0.26 ± 0.01a	0.47 ± 0.01a	0.27 ± 0.02a
ML	0.21 ± 0.01a	0.39 ± 0.02ab	0.40 ± 0.02ab
HL	0.24 ± 0.01a	0.33 ± 0.02b	0.43 ± 0.02b
Q	0.24 ± 0.01a	0.37 ± 0.02a	0.39 ± 0.03a
Q + P	0.25 ± 0.01a	0.38 ± 0.02a	0.37 ± 0.03a
Q + P + L	0.23 ± 0.01a	0.42 ± 0.03a	0.35 ± 0.03a
Q + L	0.23 ± 0.01a	0.41 ± 0.02a	0.36 ± 0.02a

The same letters after SE in columns indicate that the mean values do not differ significantly among light treatments or among seedlings’ combinations in Tukey’s test at *P* < 0.05

LL, low light (10% of full sun light); ML, medium light (25% of full sun light); HL, high light (100% of full sun light); P, three *Prunus serotina* seedlings; P + Q, six *P. serotina* seedlings in competition with three *Q. petraea* seedlings; P + Q + L, six *P. serotina* seedlings in competition with three *Q. petraea* seedlings with mulching with *P. serotina* leaves; Q, three *Quercus petraea* seedlings; Q + P, three *Quercus petraea* seedlings in competition with six *P. serotina* seedlings; Q + P + L, three *Q. petraea* seedlings in competition with six *P. serotina* seedlings with mulching with *P. serotina* leaves; Q + L, three *Q. petraea* seedlings with mulching with *P. serotina* leaves

### Light treatments and meteorological conditions

Air temperature and relative humidity (RH) were monitored with HOBO Pro v2 (OnSet Computers, Pocasset, MA, USA) throughout the growing season. Six HOBOs (two per light treatment) were fixed 80 cm above the ground and registered data every 20 min. Microclimatic differences were most noticeable between shade treatments and HL. Shading decreased monthly mean temperatures, monthly amplitudes and increased relative humidity (RH) compared with HL. The coldest months were May and September (15.87 ± 0.15, 13.86 ± 0.11 °C), the hottest was August (17.97 ± 0.12 °C). The differences between HL and ML in monthly mean temperature were 1.62 °C in June, 1.71 °C in August, and 0.97 °C in September. The lowest (1.34 °C in September) and the highest temperature (35.90 °C in August) values were observed in HL.

### Light curves of chlorophyll *a* fluorescence

Chlorophyll *a* fluorescence was measured in August 2012 using a Fluorescence Monitoring System (FMS 2, Hansatech, Norfolk, UK) operating in online mode. Six seedlings per species and light treatment (two per block) were randomly chosen and dark-treated for 30 min. in the laboratory prior to measurements of minimal (F_0_) and maximal fluorescence (F_m_) (Maxwell and Johnson 2000). Each leaf was exposed to modulated measuring light at 0.05 µmol m^−2^ s^−1^. After reading F_0_, a saturating 0.7 s pulse of light (PPF = 15,300 µmol quanta m^−2^ s^−1^, PPF – photosynthetic photon flux) was switched on to induce F_m_. Maximum quantum yield of PSII photochemistry was calculated using to the formula F_v_/F_m_, where variable fluorescence F_v_ = F_m_ − F_0_. To generate light response curves of PSII quantum yield (Φ_PSII_ = F_m_′ − F_s_/F_m_′; F_m_′—maximal fluorescence in the light, F_s_—steady state fluorescence), the leaf in the clip was illuminated with actinic light of increasing intensity (0, 12, 38, 90, 175, 295, 454, 653, 894, 1177, 1503 µmol m^−2^ s^−1^). For each light level, after a stable steady state fluorescence (F_s_) was reached, a 0.7 s saturating pulse was delivered and maximum light-adapted fluorescence (F′_m_) was determined (Rascher et al. 2000). Quantum yield of PSII was calculated according to the methods of Genty et al. (1989).

### Mathematical model of leaf absorbed light energy partitioning

Hendrickson et al. (2004) developed a method to quantify the fate of light energy absorbed by leaves. The following equations were applied to calculate quantum yield of PSII photochemistry (Φ_PSII_), quantum yield of thermal energy dissipation (Φ_NPQ_), and quantum yield of constitutive fluorescence and thermal dissipation (Φ_f,D_), respectively:

1 $${{{\varvec{\Phi}}}_{{\text{PSII}}}}={\text{F}}_{{\text{m}}}^{\prime } - {{\text{F}}_{\text{s}}}/{\text{F}}_{{\text{m}}}^{\prime }$$

2 $${{{\varvec{\Phi}}}_{{\text{NPQ}}}}={{\text{F}}_{\text{s}}}/{\text{F}}_{{\text{m}}}^{\prime } - {{\text{F}}_{\text{s}}}/{{\text{F}}_{\text{m}}}$$

3 $${{{\varvec{\Phi}}}_{{\text{f}},{\text{D}}}}={{\text{F}}_{\text{s}}}/{{\text{F}}_{\text{m}}}$$

To estimate the fractions of energy partitioned to the three processes (Φ_PSII_, Φ_NPQ_, Φ_f,D_) the areas below curves representing relationships between PPF of fluorescence induction and respective energy fraction were calculated as sums of products:

$$\sum\limits_{{{\text{PPF(n)-PPF(n-1)}}}}^{{{\text{1503}}}} {{\text{PPF}} \times (\Phi {\text{PSII or }}\Phi {\text{NPQ or }}\Phi {\text{f, D)}}} ,$$

where n—subsequent numbers of the actinic light intensity values used to induce chlorophyll *a* fluorescence.

### Gas exchange

Methods of gas exchange measurements on *P. serotina* and *Q. petraea* have been previously described in Robakowski et al. (2016). In brief, gas exchange was measured on four occasions: in June, August, September and October using the gas exchange analyzer LCA-4 (ADC, Ltd., Hoddesdon, UK). The broadleaf chamber (PLC4B) was used, with chamber conditions as follows: CO_2_ concentration in inlet air of 380 µmol mol^−1^, leaf temperature of 26 to 27 °C, and relative humidity of approximately 55%. Photosynthetic rates were measured at the saturation PPF = 1200 µmol m^−2^ s^−1^. Prior to gas exchange measurements a south-facing leaf was selected from the upper crown. Each leaf was given 20 to 30 min. to acclimate to the leaf chamber conditions prior to measuring. The window of the leaf chamber was darkened and dark respiration (R_d_, µmol CO_2_ m^−2^ s^−1^) was measured for 15 min, after which the lamp was switched on and photosynthetic rates were measured for 30 min. Five values from the stable phase of photosynthesis were averaged to obtain maximum net assimilation rate (A_max_, nmol CO_2_ g^−1^ s^−1^). Water use efficiency (WUE, µmol CO_2_ mmol^−1^ H_2_O) was calculated as the ratio of area-based maximum photosynthesis (µmol CO_2_ m^−2^ s^−1^) to transpiration rate (E, mmol H_2_O m^−2^ s^−1^). Nitrogen concentration was determined from the same leaves as those used for net CO_2_ assimilation rate to calculate the photosynthetic nitrogen use efficiency (PNUE, µmol CO_2_ mol N^−1^ s^−1^). Projected leaf area and dry mass used for measurements of gas exchange were determined to calculate leaf mass-to-area index (LMA, g m^−2^) and to calculate the net carbon gain of foliage for each individual (A_crown_, nmol CO_2_ g^−1^ s^−1^) by multiplying mass-based photosynthetic rates by the total leaf mass of individual seedlings. Due to the time-consuming nature of gas exchange measurements we were unable to measure these traits on all treatments, and excluded the P + Q (and Q + P) treatment combinations.

### Leaf nitrogen content analyses

Total nitrogen content was determined using the Kjeldahl method. The digestion of leaf samples was conducted using a digestion system with sulfuric acid at 420 °C (Foss Tecator). Nitrogen was determined by distillation with water vapour in the apparatus of Parnas-Wagner. Leaf nitrogen content was recalculated per leaf mass and area using LMA.

### Leaf area and dry mass of seedlings

Three seedlings per species, light treatment, competition/mulching combination and block were used for the biomass allocation analyses. Leaves were scanned and total leaf area was determined with the program DigiShape (Cortex Nova, Poland). Plant organs were then dried at 65 °C for 48 h and weighed for dry mass. Relative growth rate (RGR) was calculated according to the formula (Hunt 1982): ${\text{RGR}}=\frac{{{\text{ln}}({{\text{W}}_2}) - {\text{ln}}({{\text{W}}_1})}}{{{{\text{t}}_2} - {{\text{t}}_1}}}$, where W_1_—mean initial total seedling dry mass calculated using ten seedlings prior to the experiment, W_2_—total seedling dry mass at the end of the experiment, t_1_—day of year 146 (May), t_2_—day of year 269 (October). Leaf area ratio (LAR) was calculated with the formula: LAR = A_L_/W_2_, where A_L_ – total seedling leaf area.

### Data analyses

Prior to the analysis of variance the gas exchange data were logarithmically transformed or function $z=arcsin\sqrt p ~$was applied to the data in the form of fractions or percentages to obtain a normal distribution. The effects of block, sampling date, light, combination and their interactions on LMA and photosynthetic parameters of *P. serotina* and *Q. petraea* leaves were analyzed using ANOVA in split–split plot design. ANOVA in split-plot design was also applied to compare the effects of block, light and combination on fractions of energy partitioned to the three processes (Φ_PSII_, Φ_NPQ_, Φ_f,D_). Analyses were conducted for energy fractions calculated as both the areas beneath the light curves and the values of energy fractions at PPF = 295 µmol m^−2^ s^−1^. This value of fluorescence induction PPF was chosen because net CO_2_ assimilation rates were not light-saturated at this light level and there was no photoinhibition in LL-acclimated seedlings.

For each study species, a two-way ANOVA with interactions was used to compare mean values of RGR among light treatments and competition and mulching combinations. Prior to analyses, RGR values were transformed with $z=arcsin\sqrt p$. A linear regression was used to examine interspecific differences in biomass allocation to leaves vs. roots between the study species. All statistical analyses were conducted using Statistica 12.0 (StatSoft, Inc., USA) and Sigmaplot 13.0 (Systat Software, Inc., USA).

## Results

### Partitioning of energy absorbed by leaves

**Fig. 1:**
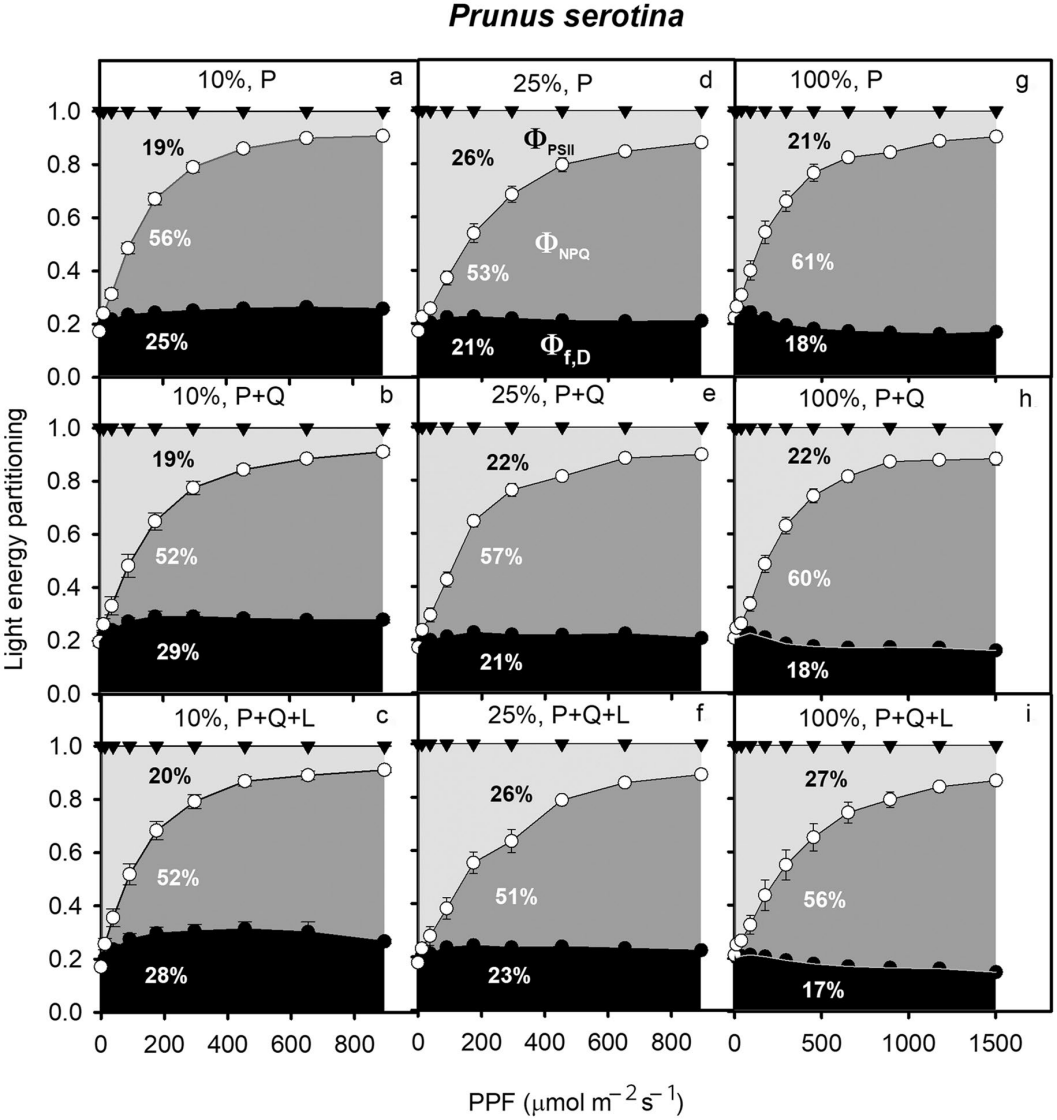
Partitioning of light energy (shown as the percent area under the curve) absorbed by leaves of *Prunus serotina* seedlings acclimated to 10 (**a**–**c**), 25 (**d**–**f**) or 100% of full sun light (**g**–**i**) and growing in one of three combinations: P, three *P. serotina* seedlings; P + Q, six *P. serotina* and three *Quercus petraea* seedlings; P + Q + L, six *P. serotina* and three *Q. petraea* seedlings with mulching with *P. serotina* leaves; Φ_PSII_, quantum yield of PSII photochemistry Φ_PSII_; Φ_NPQ_, quantum yield of ΔpH- and xanthophyll-regulated thermal energy dissipation; Φ_f,D_, quantum yield of constitutive fluorescence and thermal energy dissipation. Triangles—Φ_PSII_ + Φ_NPQ_ + Φ_f,D_; white circles—Φ_NPQ_ + Φ_f,D_; black circles – Φ_f,D_ (mean ± SE). Light green area—a fraction of energy transferred to photochemistry; dark green area—energy dissipated as heat; black area—constitutive energy losses (*n* = 6 seedlings per species, light treatment and combination)

**Fig. 2:**
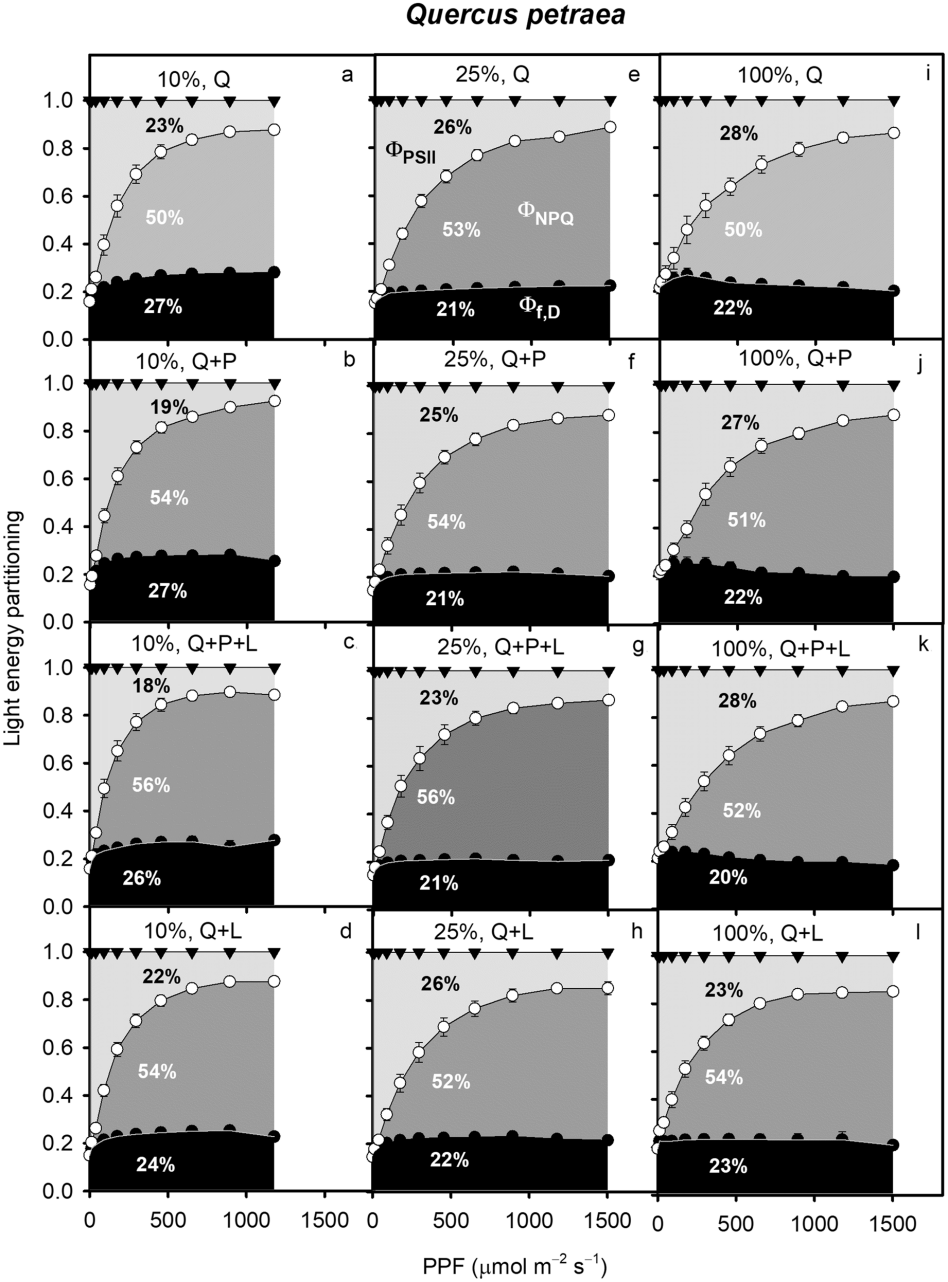
Partitioning of light energy absorbed by leaves of *Quercus petraea* seedlings acclimated to 10 (**a**–**c, d**), 25 (**d**–**g**) or 100% of full sun light (**g**–**j**) and growing in one of four combinations: Q, three *Q. petraea* seedlings; Q + P, three *Quercus petraea* seedlings and six *P. serotina* seedlings; Q + P + L, three *Quercus petraea* seedlings and six *P. serotina* with mulching with *P. serotina* leaves; Q + L, three *Q. petraea* seedlings with mulching. For the further explanations, see Fig. 1 (*n* = 6)

In LL, both study species exhibited Φ_PSII_ of approximately 20% of total leaf absorbed light energy, which increased to 27–28% in HL (*P. serotina: F*_2_ = 47.3, *P* = 0.002, Fig. 1, Q. *petraea: F*_2_ = 6.1, *P* = 0.004, Fig. 2). In Figs. 1 and 2, the percent values indicates the percent area under the curve. *P. serotina* increased Φ_NPQ_ from LL to HL, but this was not observed in *Q. petraea* (Figs. 1, 2). Interestingly, under HL, Φ_PSII_ was 6% higher for *P. serotina* in competition with *Q. petraea* and mulching (P + Q + L) than in *P. serotina* monoculture (P) suggesting that the interspecific competition may stimulate an increase in Φ_PSII_ of this invasive species (Fig. 1g, i).

In *P. serotina*, Φ_f,D_ decreased with higher growth light intensity (Fig. 1). In the P treatment, Φ_f,D_ was 7% higher in LL than in HL (Fig. 1a, g). For P + Q + L and for *P. serotina* in competition with *Q. petraea* without mulching (P + Q), Φ_f,D_ was 11% higher in LL than in HL (Fig. 1b, c, h, i). The LL seedlings of *Q. petraea* showed the highest Φ_f,D_ in all treatments, with ML and HL being lower and not significantly different from each other (Fig. 2).

Φ_f,D_, Φ_NPQ_ and Φ_PSII_ were also compared among the light treatments and species combinations at PPF = 295 µmol m^−2^ s^−1^. The invasive seedlings in P + Q + L showed higher Φ_PSII_ than those growing in P or P + Q (Table 1), providing some support for hypothesis 1. On average, Φ_NPQ_ decreased and Φ_PSII_ increased as light availability increased (Tables 1, S1). Φ_NPQ_ was lower in P + Q + L than in P and P + Q (*F*_2_ = 6.2, *P* = 0.005). In HL, consistent with hypothesis 3, competition and allelopathic effects led to more significant differences in Φ_PSII_ than those observed in ML and LL (Fig. 1). Similar to Φ_f,D_ expressed as the area beneath the curve, Φ_f,D_ of *P. serotina* seedlings at 295 PPF averaged across all treatments was highest in seedlings growing in LL, whereas those in ML and HL were significantly lower and did not differ from each other based on a Tukey’s test (Table 1).

In *Q. petraea*, Φ_f,D_ did not differ among the light treatments (Table 1, S1, Fig. 2). On average, *Q. petraea* seedlings showed light acclimation of Φ_NPQ_ and Φ_PSII_ (Table 1). At the induction PPF = 295 µmol m^−2^ s^−1^, Φ_NPQ_ was significantly higher in LL than in HL, while the inverse was true for Φ_PSII_ (Table 1, S1). Consistent with hypothesis 1, competition and mulching treatments of *P. serotina* leaves had no effect on energy partitioning of native *Q. petraea* seedlings which showed lower plasticity in light energy partitioning compared with *P. serotina*.

### Leaf structure

From the beginning to the end of the experiment, LMA of *P. serotina* increased by 30% and was not influenced by competition or mulching (Tables 2, S2). LMA did not differ between LL and ML, but increased by 29% in the HL treatment (Table S2).

**Table 2:** Analysis of variance in split–split plot design of block, date of sampling, light and treatment combinations and interactions on leaf mass to area ratio (LMA, g m^−2^), net CO_2_ assimilation rate at saturating light (A_max_, nmol g^−1^ s^−1^), leaf dark respiration (R_d_, nmol g^−1^ s^−1^), photosynthetic nitrogen use efficiency (PNUE, µmol CO_2_ mol N^−1^), water use efficiency (WUE, µmol CO_2_ mmol H_2_O^−1^), and respiratory costs of photosynthesis (R_d_/A_max_) of *Prunus serotina* and *Quercus petraea* seedlings. The block effect was not significant, therefore the effects of block and of interactions with block are omitted

Effect	*Prunus serotina*			*Quercus petraea*		
	*df*	*F*	*P*	*df*	*F*	*P*
LMA						
Date of sampling	3	49.32	< 0.001	3	12.80	0.007
Light	2	94.95	< 0.001	2	122.32	< 0.001
Date of sampling × light	6	6.49	0.001	6	1.94	0.135
Combination	1	0.22	0.644	2	5.54	0.007
Date × combination	3	1.91	0.155	6	0.781	0.589
Light × combination	2	4.38	0.024	4	3.05	0.026
Date × light × combination	6	0.81	0.575	12	0.657	0.783
A_net_						
Date of sampling	3	1.78	0.251	3	15.60	0.003
Light	2	25.59	0.005	2	131.28	< 0.001
Date of sampling × light	6	0.31	0.748	6	3.76	0.016
Combination	1	10.01	0.004	2	4.05	0.024
Date × combination	3	2.65	0.072	6	1.34	0.259
Light × combination	2	2.99	0.069	4	2.58	0.049
Date × light × combination	6	0.94	0.484	12	0.97	0.493
R_d_						
Date of sampling	3	6.37	0.030	3	0.290	0.832
Light	2	26.84	< 0.001	2	13.72	0.006
Date of sampling × light	6	3.30	0.026	6	2.24	0.093
Combination	1	0.35	0.561	2	6.37	0.004
Date × combination	3	0.87	0.473	6	0.63	0.451
Light × combination	2	0.39	0.679	4	0.937	0.451
Date × light × combination	6	1.35	0.276	12	1.20	0.315
PNUE						
Date of sampling	3	3.79	0.090	3	57.25	< 0.001
Light	2	21.36	0.002	2	84.65	< 0.001
Date of sampling × light	6	3.84	0.015	6	4.77	0.006
Combination	1	11.35	0.003	2	2.77	0.072
Date × combination	3	2.53	0.081	6	0.66	0.685
Light × combination	2	1.54	0.234	4	3.24	0.020
Date × light × combination	6	0.17	0.982	12	0.58	0.844
WUE						
Date of sampling	3	21.42	0.001	3	14.19	0.005
Light	2	13.89	0.006	2	43.37	< 0.001
Date of sampling × light	6	3.50	0.020	6	3.97	0.013
Combination	1	1.12	0.300	2	6.31	0.004
Date × combination	3	0.54	0.658	6	1.15	0.347
Light × combination	2	0.68	0.515	4	1.94	0.119
Date × light × combination	6	2.50	0.510	12	1.56	0.137
R_d_/A_max_						
Date of sampling	3	1.74	0.258	3	3.28	0.109
Light	2	0.95	0.443	2	44.23	< 0.001
Date of sampling × light	6	2.28	0.088	6	4.10	0.011
Combination	1	3.56	0.071	2	0.36	0.698
Date × combination	3	0.27	0.849	6	0.48	0.822
Light × combination	2	1.15	0.334	4	4.69	0.003
Date × light × combination	6	0.37	0.894	12	0.99	0.470

**Fig. 3:**
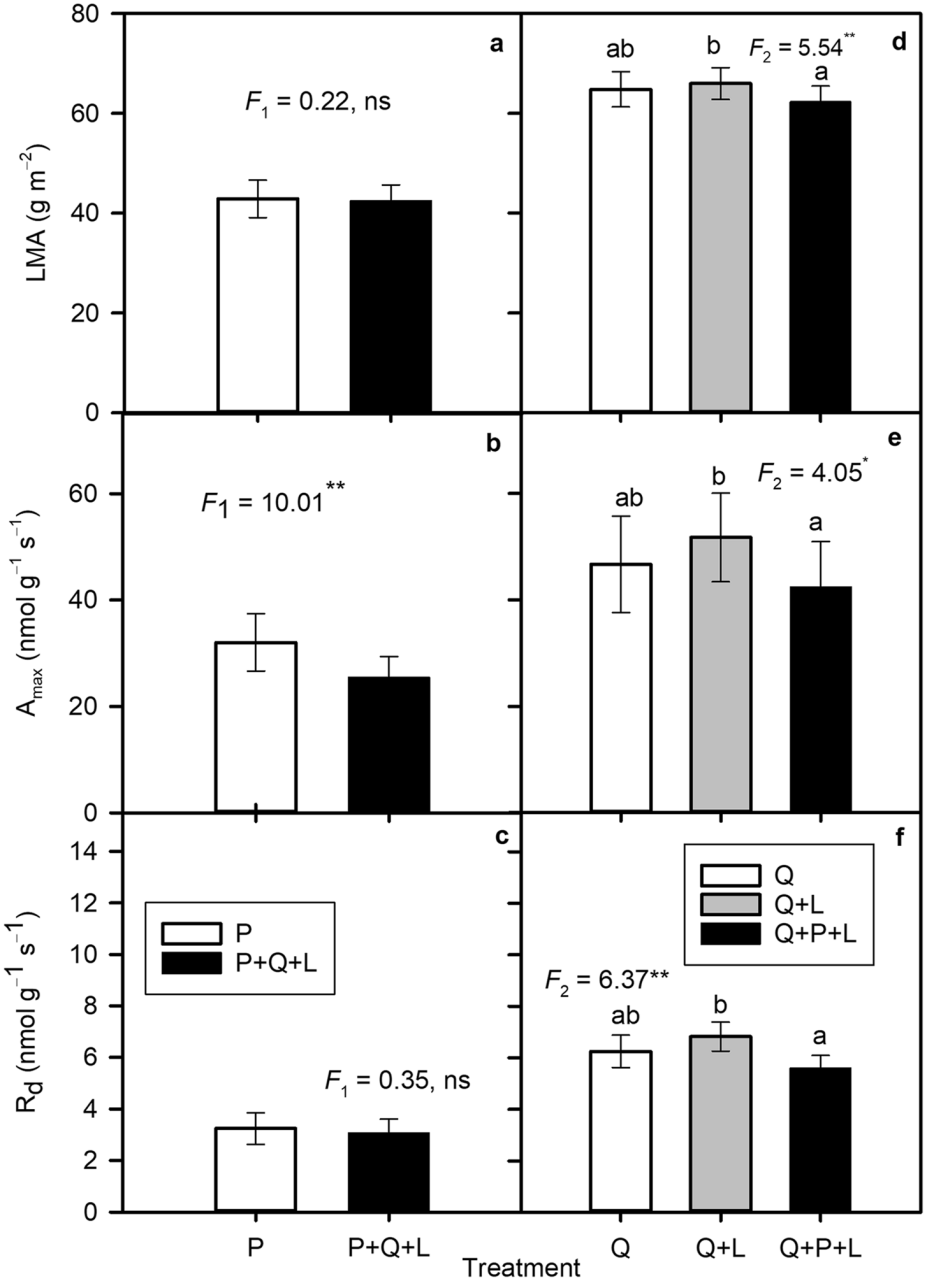
*Left hand panels* The mean (± SE) values of **a** leaf mass-to-area ratio (*LMA*), **b** maximum net CO_2_ assimilation rate expressed per leaf dry mass (A_max_), and **c** dark respiration rate per unit dry mass (R_d_) of *P. serotina* seedling monocultures (P) or six *P. serotina* seedlings in competition with three *Q. petraea* seedlings with mulching (P + Q + L). *Right hand panels* The mean (± SE) values of **d** *LMA*, **e** A_max_, and **f** R_d_ of *Q. petraea* seedling monocultures (Q), three *Q. petraea* seedlings with mulching (Q + L), or three *Q. petraea* seedlings in competition with six *P. serotina* seedlings with mulching (Q + P + L). All data were pooled across June, August, September and October. The *F* values with the number of degrees of freedom in lower index and probability obtained from ANOVA are shown. 0.05 > P* ≥ 0.01, 0.01 > P** ≥ 0.001, P*** < 0.001 (*n* = 36)

LMA of *Q. petraea* increased by approximately 15% from June to July, then remained relatively unchanged through the remainder of the growing season (Tables 2, S2). Compared to LL, LMA of *Q. petraea* was 4% higher in ML and 19.5% higher in HL (Table S2). When all data were pooled across all the sampling dates, mulching with *P. serotina* leaves (Q + L) increased LMA of *Q. petraea* compared to seedlings grown in monoculture (Q) which suggested a positive nutritional effect contrary to hypothesis 4, but LMA of the interspecific competition and mulching treatment (Q + P + L) was similar to Q (Fig. 3d). Differences among the treatment combinations were further modified by light conditions of growth. LMA of *Q. petraea* was similar in all competition and mulching treatments in HL, but differences among treatments became more pronounced as light limitations increased (Table S2).

### Photosynthetic capacity

In *P. serotina*, A_max_ expressed per gram leaf dry mass did not change considerably from month to month and was 28 nmol g^−1^ s^−1^ on average. In contrast, A_max_ of *Q. petraea* decreased progressively from June to September from 62 to 32 nmol g^−1^ s^−1^ and its mean seasonal value was 47 nmol g^−1^ s^−1^ (Tables 2, S3, S4). In *P. serotina*, A_max_ was twofold higher in HL than in LL and this was accompanied by twofold increase in R_d_ (Table S3). In *Q. petraea*, A_max_ was 2.5-fold higher in HL than in LL, and R_d_ increased by 30% (Table S4).

When the mean values calculated across all the sampling dates were compared, A_max_ in P + Q + L decreased compared with P, while R_d_ remained stable, rejecting hypothesis 2 (Table 2; Fig. 3b, c). The Q + L treatment exhibited the highest rates of both A_max_ and R_d_, while the Q + P + L treatment was similar to the monoculture (Tables S3, S4, Fig. 3e, f). Additionally, the light × treatment combination interaction influenced A_max_ of *Quercus*, indicating a positive effect of mulching that was the most significant in LL (Table S4).

In contrast to A_max_, when A_crown_ was calculated for individual seedlings, it was higher on average in *P. serotina* than in *Q. petraea* (Tables S5, S6). Thus, while hypothesis 2 was rejected for *P. serotina* leaf level traits, the invasive species did have enhanced photosynthetic capacity at the crown level. A_crown_ of *P. serotina* increased abruptly in September (DOY—day of year 269) (due to the high leaf mass and area per seedling), whereas A_crown_ of *Q. petraea* did not change significantly across the season. The most striking difference between the two species was their response to higher levels of light availability: invasive *P. serotina* increased A_crown_ 40-fold from LL to HL, while native *Q. petraea* only increased fourfold (Table S6).

### Photosynthetic efficiency

**Fig. 4:**
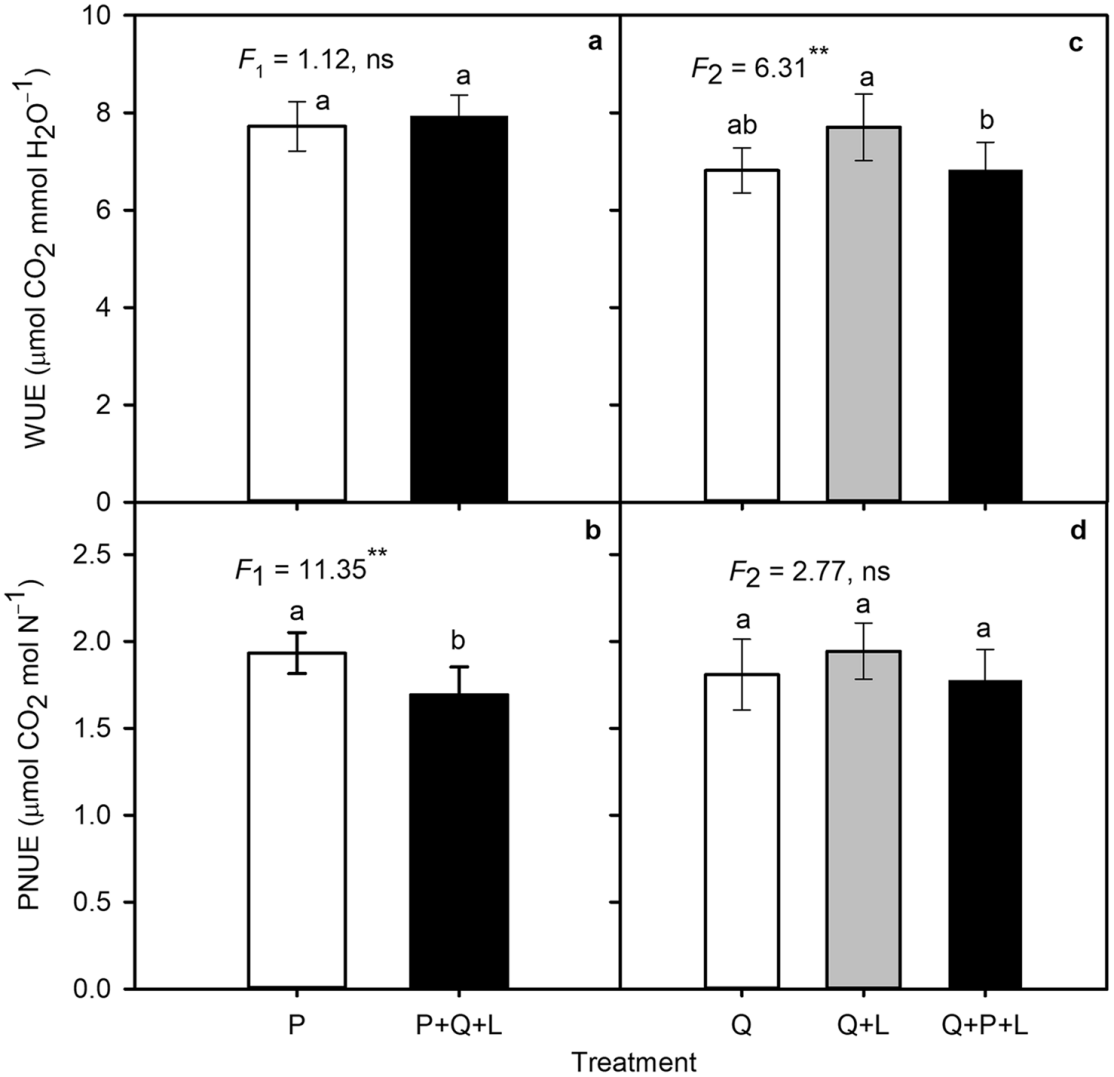
*Left hand panels* The mean (± SE) values of **a** photosynthetic water use efficiency (WUE) and **b** photosynthetic nitrogen use efficiency (PNUE) of *P. serotina* seedling monocultures (P) or six *P. serotina* seedlings in competition with three *Q. petraea* seedlings with mulching (P + Q + L). *Right hand panels* **c** WUE and **d** PNUE of *Q. petraea* seedling monocultures (Q), three *Q. petraea* seedlings with mulching (Q + L), or three *Q. petraea* seedlings in competition with six *P. serotina* seedlings with mulching (Q + P + L). All data were pooled across June, August, September and October. The *F* values with the number of degrees of freedom in lower index and probability obtained from ANOVA are shown. 0.05 > P* ≥ 0.01, 0.01 > P** ≥ 0.001, P*** < 0.001 (*n* = 36)

In both species, photosynthetic nitrogen use efficiency (PNUE) decreased throughout the growing season, but was always higher in *P. serotina* than in *Q. petraea*, consistent with hypothesis 2. Both invasive *P. serotina* and native *Q. petraea* increased PNUE on average in ML and HL compared with LL (Tables 2, S7, S8). *P. serotina* was able to enhance PNUE by 54% and *Q. petraea* by 83% in HL compared with LL. P + Q + L showed a 24% lower PNUE compared to P (Table S7, Fig. 4b), while PNUE of *Q. petraea* was similar in all competition and mulching treatments (Table 2; Fig. 4d). Leaf nitrogen concentration decreased with increasing light and LMA (*Q. petraea* LL 30.12 ± 0.60 and HL 27.33 ± 0.74 mg g^−1^; *P. serotina* LL 33.19 ± 0.91 and HL 29.33 ± 1.19 mg g^−1^). In *Q. petraea*, the highest leaf nitrogen concentration was in Q + L (30.69 ± 0.74 mg g^−1^), and in *P. serotina* it decreased from 33.96 ± 0.89 in P to 30.53 ± 1.11 mg g^−1^ in P + Q + L.

Photosynthetic water use efficiency (WUE) in both species depended on date of sampling, light and, in *Q. petraea*, on treatment combination (Table 2). On average in *P. serotina* seedlings, WUE increased from May (DOY 146) to September (DOY 269) and the opposite trend was observed in *Q. petraea* (Tables S7, S8). In both species, WUE was greatest in HL and declined as light became more limiting. Interspecific competition and mulching treatments did not affect WUE of *P. serotina* (Table S7, Fig. 4a), while WUE increased significantly in the Q + L treatment as compared to Q and Q + P + L (Table S8, Fig. 4c).

The overall respiration costs of photosynthesis (R_d_/A_max_) were higher in *Q. petraea* compared with *P. serotina* (R_d_ /A_max_ = 0.17 ± 0.01 and 0.12 ± 0.01, respectively), supporting hypothesis 2. This was true throughout the growing season, with differences between species being most extreme at the end of the growing season. Date of sampling, light environment and treatment combination did not significantly affect R_d_/A_max_ in *P. serotina* (Table 2). In contrast, light environment significantly affected respiration costs of photosynthesis in *Q. petraea*, which decreased as light availability increased (LL = 0.23 ± 0.02, ML = 0.17 ± 0.02, HL = 0.12 ± 0.01). In ML, R_d_/A_max_ was similar in Q and Q + L, and lowest in Q + P + L (0.17 ± 0.03, 0.21 ± 0.04 and 0.14 ± 0.03, respectively).

### Relative growth rate and biomass allocation to foliage

**Fig. 5:**
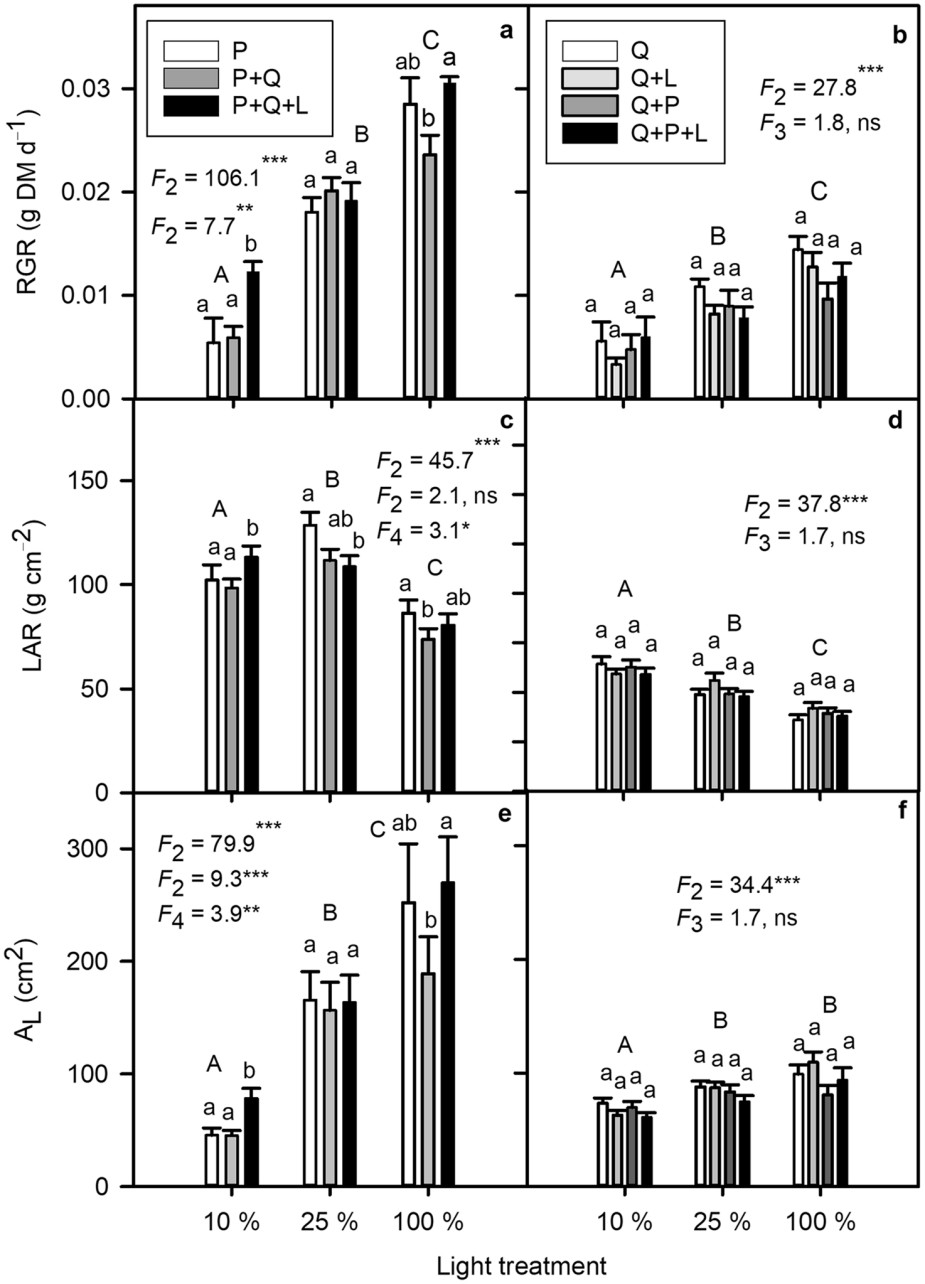
Relative growth rate (RGR), leaf area ratio (LAR), and total leaf area (A_L_) of *Prunus serotina* (**a, c, e**) and *Quercus petraea* (**b, d, f**) seedlings which were grown in one of three light treatment (10, 25 or 100% of full sun light) and five combinations: P, Q, Q + L, P + Q (Q + P) or P + Q + L (Q + P + L). The *F* values with the number of degrees of freedom in lower index and probability obtained from two-factorial analysis of variance with light, combination and interaction are shown. The same capital letters indicate that the mean values do not significantly differ among light treatments. The same small letters indicate that the mean values are not significantly different between combination within a light treatment in Least Significant Difference test. 0.05 > P* ≥ 0.01, 0.01 > P** ≥ 0.001, P*** < 0.001 (*Q. petraea n* = 76, *P. serotina n* = 63)

**Fig. 6:**
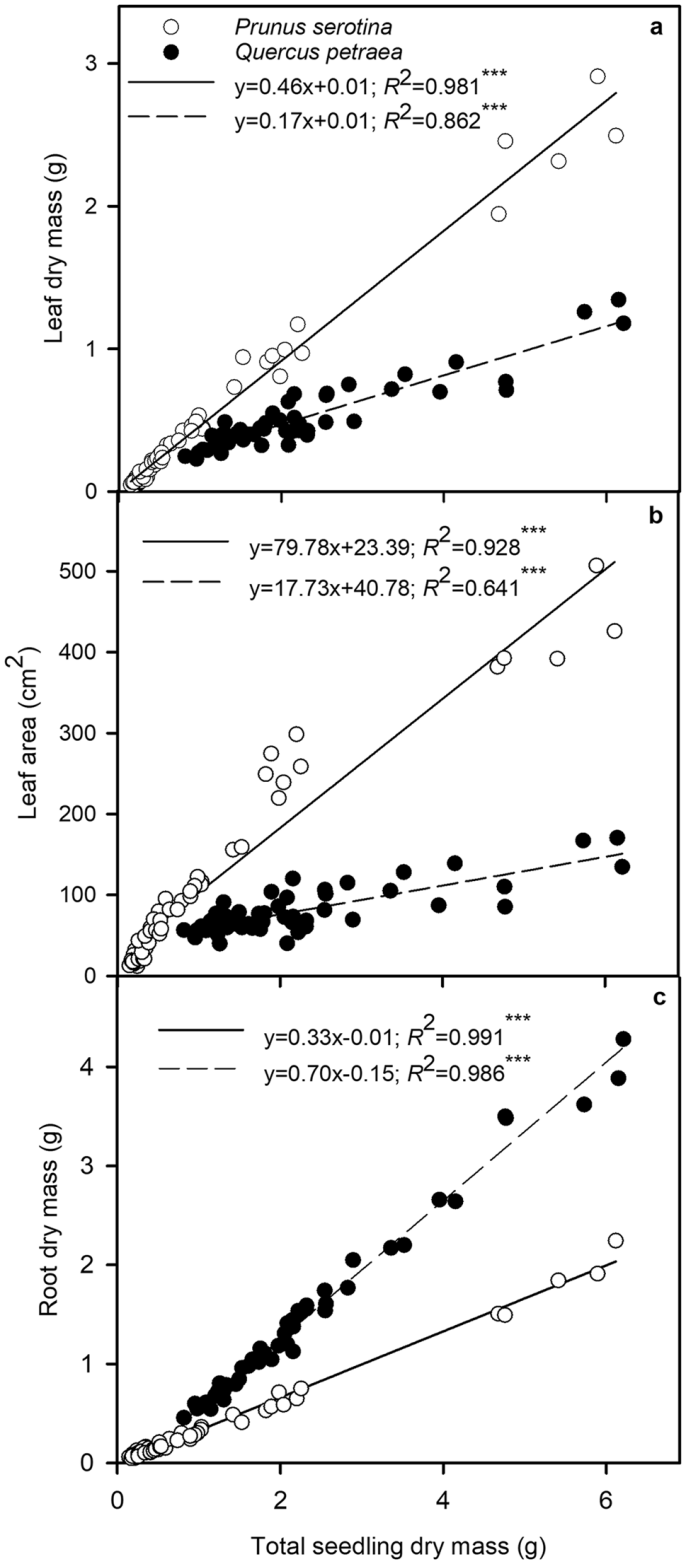
Linear regressions between **a** leaf dry mass, **b** leaf area, and **c** root dry mass and total seedling dry mass. The seedlings were grown in one of three light treatment (10, 25 or 100% of full sun light) and three combination treatments [P, Q or P + Q + L (Q + P + L)]. Each point represents the mean value of three seedlings per species per pot (*n* = 216 seedlings per species)

Mean initial dry mass (DM) of *P. serotina* and *Q. petraea* organs were: roots 0.190 ± 0.052, 0.357 ± 0.031, shoots: 0.069 ± 0.005, 0.129 ± 0.020, and leaves: 0.034 ± 0.003, 0.415 ± 0.052 g, respectively (*n* = 10, *n*—number of seedlings). Initial total seedling DM of *Q. petraea* was 0.901 ± 0.099 and *P. serotina* 0.293 ± 0.056 g. *P. serotina* had higher RGR than *Q. petraea* (0.133 ± 0.004 and 0.09 ± 0.004 g total DM day^−1^, respectively, all data pooled, *F*_1_ = 48.6, *P* < 0.0001) and the differences between the species increased as light availability increased: compared to *Q. petraea*, RGR of *P. serotina* 1.8-fold higher in LL, 2.1-fold higher in ML, and 2.3-fold higher in HL, providing support for hypotheses 3 and 5 (Figs. 5, 6). RGR of *Q. petraea* did not differ significantly among competition and mulching treatments (Fig. 5b), while RGR of P + Q + L in low light was greater than that of P and P + Q (Fig. 5a). Leaf area ratio (LAR) decreased and total leaf area (A_L_) increased with light for both species, but more significantly for the invasive than native species (Fig. 5c–f). In LL, RGR, LAR and A_L_ were highest in P + Q, and in HL, RGR and A_L_ were lower in P + Q than in P + Q + L (Fig. 1a, c, e).

The linear relationship between *P. serotina* total seedling dry mass vs. leaf dry mass had over a two-fold steeper slope than for *Q. petraea* seedlings (Fig. 6a), providing support for hypothesis 5. The slope of the relationship between the total seedling dry mass vs. leaf area was more than fourfold steeper for *P. serotina* compared with the slope for *Q. petraea* (Fig. 6b). Alternatively, the slope of the relationship between total seedling dry mass vs. root dry mass was significantly steeper for *Q. petraea* than for *P. serotina* (Fig. 6c).

## Discussion

### Leaf absorbed light energy partitioning

Competition between invasive *P. serotina* and native *Q. petraea* has been observed *in situ* under the canopy of Scots pine forests in Europe. We simulated this competition and potential allelochemical interspecific interactions under different controlled light environments. This study complements and adds to prior studies comparing morphological and physiological traits of invasive and native species (e.g. Boyd et al. 2009; Funk 2008; Harrington et al. 1989; Mangla et al. 2011; Molina-Montenegro et al. 2012; Leishman et al. 2007; van Kleunen et al. 2010), and focuses on species responses to seasonality, light environment, competition, and allelopathic effects from mulching with *P. serotina* leaves. Our results show that the acclimation of leaf absorbed light energy partitioning is more strongly affected by light environment than by competition and allelopathy (Tables 1, S1; Figs. 1, 2). Here, we expand on prior studies by focusing on interspecific competition for light and its effect on partitioning of light energy into three competing processes: Φ_PSII_, Φ_NPQ_, and Φ_f,D_. It is worth noting that *P. serotina* increased Φ_NPQ_ and Φ_PSII_ markedly at the expense of Φ_f,D_ when growing in ML and HL compared with LL, especially when in competition with *Q. petraea* with mulching (Fig. 1c, f, i). This response of invasive *P. serotina* to light can be regarded as the ‘Oskar Syndrome’ at the bioenergetics level. The ‘Oskar Syndrome’ is a dynamic increase in the growth of a shade-acclimated plant following exposure to a brighter light environment (Closset-Kopp et al. 2007, 2011). Higher amounts of leaf absorbed light energy translocated to Φ_PSII_ can contribute to an increase in growth under optimal light conditions. *Q. petraea* also increased Φ_NPQ_ and Φ_PSII_ in HL compared with LL, but the differences between the light treatments were less extreme than those observed for *P. serotina* (Fig. 2). For the first time, we have shown that higher plasticity of light energy partitioning can give an important competitive advantage to invasive over native species (Tables 1, S1, Figs. 1, 2). Our results also add to the body of evidence showing higher morphological plasticity of invasive compared to native species in response to light (Figs. 5, 6; Daehler 2003; Funk 2008; Molina-Montenegro 2012). In contrast to our results, Demmig-Adams and Adams (1996) observed a similar conversion state of the xanthophyll cycle and a similar level of energy dissipation for a given degree of light stress, independent of species or light stress conditions.

Interestingly, in our study, at PPF = 295 µmol m^−2^ s^−1^, *P. serotina* increased Φ_PSII_ growing in the presence of its competitor with mulching (P + Q + L vs. P). Together with a lack of difference between P and P + Q these results suggest that there was a positive allelopathic effect of mulching, or a positive synergistic effect of interspecific competition and allelopathy on *P. serotina* Φ_PSII_. This is in agreement with hypothesis 1, suggesting that invasive species can enhance photosynthetic performance by transferring more energy to photochemistry when growing in the presence of a competitor (Tables 1, S1). An increase in Φ_PSII_ may have occured due to higher availability and allocation of nitrogen to photochemistry in P + Q + L. However, our results do not support hypothesis 2 since A_max_ was lower in P + Q + L compared to P, suggesting that energy was dissipated as heat or used in non-photosynthetic processes (Niyogi 1999). Nevertheless, our results suggest that the responsiveness of invasive *P. serotina* to light and competition with or without mulching is more dynamic than that of native *Q. petraea* (Tables 1, S1, Fig. 5). Compared to observed levels of Φ_NPQ_ obtained in our study (*P. serotina* from 52 to 61%, *Q. petraea* from 50 to 56%), leaves of grapevines growing in full sunlight have been found to dissipate up to 75% of total absorbed daily radiation via ΔpH- and xanthophyll-regulated thermal dissipation (Hendrickson et al. 2004). Compared with our experiment, higher values of Φ_NPQ_ resulted from growing grapevines in a greenhouse. Another study observed that under drought stress, Φ_NPQ_ can be as high as 92% of total absorbed radiation (Flexas and Medrano 2002). *P. serotina* and *Q. petraea* in our study increased Φ_PSII_ in ML and HL compared with LL at the expense of Φ_f,D_. The stress caused by a deficit of light in the LL treatment enhanced Φ_f,D_ and reduced Φ_PSII_ compared with HL.

### Leaf structure

*P. serotina* had lower LMA than *Q. petraea* independent of sampling date, light environment or treatment combination (Table S4, Fig. 1a, d). The global leaf economic spectrum indicates that species with lower LMA have higher photosynthetic capacities (Wright et al. 2004). Interestingly, in our experiment the mean LMA value of *P. serotina* (43 ± 1 g m^−2^) was lower than LMA of this species in Minnesota, where it naturally occurs (48 ± 3 g m^−2^) (Sendall and Reich 2013). This observation supports the hypothesis that invasive *P. serotina* is able to decrease LMA in its introduced geographical range. In our study, both species increased LMA in HL compared with LL and ML, but contrasting with the results of Leishman et al. (2010), they did not differ substantially in the magnitude of this increase. Pattison et al. (1998) showed that five invasive and four native species grown in the shade had lower LMA than those grown in full sun, while other studies have shown that *P. serotina* juveniles growing in full sun have approximately threefold higher LMA than in the understory (Harrington et al. 1989; Sendall and Reich 2013). Leaf structural changes reflected by LMA, together with biochemical mechanisms of excess energy dissipation, allow plants to use light more efficiently for photosynthesis and protect the photosynthetic apparatus against excess energy (Ellsworth and Reich 1992; Niinemets et al. 1998).

Photosynthetic and leaf structural responses of *Q. petraea* and *P. serotina* to different light environments suggest that they are functionally similar. Both species increased A_max_, PNUE and LMA with increasing light. However, high light availability favoured *P. serotina* over *Q. petraea* (Tables S2, S6, Fig. 5). This is in accordance with Firn et al. (2010) who found that the ability of invading grasses to suppress natives was greater under higher resource supply.

In our study, LMA and A_max_ were lower for Q + P + L when compared with Q + L indicating a significant effect of interspecific competition on *Q. petraea* leaf structure and photosynthesis when the competition occurs with mulching (Tables S2, S4 Fig. 3d, e). In contrast to our results, Kozovits et al. (2005) found that the competition between two co-occurring native species in Europe *Fagus sylvatica* and *Picea abies*, was not associated with changes in LMA or carbon gain efficiency. This difference may result from more intense interspecific competition between the invasive and native species enhanced by allelopathic or nutritional effects in our experiment compared with the competition between native species in Kozovits et al. study. In HL, the mean height of *P. serotina* was 192 ± 11 mm, while the mean height of *Q. petraea* 95 ± 2 mm. *P. serotina* grew more dynamically, overtopping and shading *Q. petraea* seedlings, which caused *Q. petraea* to alter their leaf structure in response to this additional shading. In ML and HL, our invasive species benefitted directly from higher light availability for photosynthesis and indirectly by shading out the native species and effectively reducing its growth (Craine and Dybzinski 2013).

### Photosynthetic capacity and efficiency

There are conflicting results in the literature as to whether species’ gas exchange rates vary among invasive and native species and whether the difference confers growth advantages (Funk 2008). In our study, the values of LMA suggest that *P. serotina* should have higher photosynthetic capacity per unit leaf dry mass than *Q. petraea*. However, during the growing season, the invasive maintained A_max_ and R_d_ at a lower and more stable level compared with the native, rejecting hypothesis 2. Thus, our results suggest that an invasive does not need to have higher A_max_ to overtop its native competitor growing in the same niche. Our results are inconsistent with an earlier study by Leishman et al. (2007) who compared 75 native and 90 invasive plants and found that invasive species had higher A_max_ than natives, as well as another study showing that two invasive *Rubus* species had higher A_max_ compared with their native congeners (McDowell 2002). However, Boyd et al. (2009) found that the invasive *Berberis thunbergii* DC. did not differ in A_max_ from the native *Kalmia latifolia* L. and *Vaccinium corymbosum* L. Additionally, our study species *P. serotina* in its native range showed higher net CO_2_ assimilation rates than two invasive shrubs (Harrington et al. 1989). Thus, our results indicate that photosynthetic activity alone, despite its fundamental role in the acquisition of energy required for growth, does not appear to drive the invasive success of *P. serotina*.

Phenological differences in leaf flushing, senescence, and growing season length may to some extent explain differences in carbon gain and growth of the invasive and native species (Zohner and Renner 2017). *Q. petraea* emerges from acorns and begins leaf bud flushing 2–3 weeks earlier than *P. serotina*. However, the latter continues height growth for a longer period of time and under the shade of a tree canopy, and it can retain photosynthetically functional leaves further into the fall and occasionally in winter. In the natural range of *P. serotina* in Wisconsin, USA, this lengthening of the growing season does not occur, and in fact some invasives have been found to retain leaves for up to two weeks longer than *P. serotina* (Harrington et al. 1989). Thus, invasive *P. serotina* appears to use late bud flushing to avoid late spring frosts and extended leaf longevity in the fall to increase its annual carbon gain and gain a growth advantage over some native species.

In our study, the strong competitive pressure and lower A_max_ for P + Q + L resulted in decreased PNUE compared with P (Table S7, Figs. 3b, 4b). In contrast, for the native species, Q + P + L did not change A_max_ compared with Q, while Q + L showed the highest values of A_max_ compared with the other treatment combinations. Thus, the results did not confirm hypothesis 4, which stated that mulching with *P. serotina* leaves would have a negative allelopathic effect on the photosynthetic rate of *Q. petraea*. It is possible that mulching instead had positive nutritional effects on *Q. petraea*. The leaves of *P. serotina* used for mulching may have been a source of nitrogen that was utilized in photosynthetic processes and invested into photosynthetic structures, contributing to an increase in CO_2_ uptake or prunasin production used for chemical defense (Neilson et al. 2013). If prunasin from leaves used for mulching was microbiologically degraded in soil, it may also provide *Q. petraea* with nitrogen that could contribute to an increase in A_max_. However, the biochemical mechanism of this process remains unclear (Ubalua 2010).

PNUE increased with light in our experiment, but differences between light treatments were more distinguishable in *Q. petraea* (Tables S7, S8). P + Q + L reduced PNUE compared with P due to lower A_max_ and leaf nitrogen concentrations (34 mg N g^−1^ DM in P and 31 mg g^−1^ in P + Q + L) (Fig. 4b). *P. serotina* may have invested some amount of nitrogen into non-photosynthetic compounds such as prunasin for chemical defense at the expense of A_max_ (Patton et al. 1997). Other studies have observed similar trends regarding nitrogen use and chemical defense compounds; for example, a study of *Eucalytpus cladocalyx* found that there was an approximately proportional increase in cyanogenic glycoside concentration with leaf nitrogen concentration (Gleadow et al. 1998). Our results suggest that in the introduced range in Polish forests, *P. serotina* seedlings that occur en masse can assimilate and accumulate high levels of nitrogen, making it less available for *Q. petraea* (Craine and Dybzinski 2013).

Photosynthetic energy use efficiency (µmol CO_2_ (kg glucose)^−1^ s^−1^) and nitrogen use efficiency (µmol CO_2_ (g nitrogen)^−1^ s^−1^) have been found to be higher for invasive compared to native species, while costs of leaf construction are lower (Boyd et al. 2009). Our results confirmed this, as in *P. serotina*, the respiratory costs of photosynthesis were lower than in *Q. petraea* independent of sampling date, light environment or treatment combination, which supports hypothesis 2. This is consistent with prior studies showing that invasive species have lower respiratory costs compared with native species (Leishman et al. 2010; McDowell 2002; Pattison et al. 1998). Although the mean values of area- and mass-based A_max_ were lower for *P. serotina*, A_crown_ per individual *P. serotina* seedling was higher than that of *Q. petraea* (Tables S5, S6), confirming hypothesis 2. Moreover, A_crown_ of *Q. peteaea* was also competitively suppressed by the presence of the invasive in the Q + P + L treatment as compared to Q + L (Table S6).

The invasiveness of *P. serotina* was further enhanced when seedlings were exposed to HL, which was reflected by a more significant increase in A_crown_ compared with the native and more conservative *Q. petraea*.

### Biomass allocation

*P. serotina* allocated more biomass to leaves and had higher LAR, A_L_ and RGR, which conveyed an advantage in the competition for light compared with *Q. petraea* (Figs. 5, 6). This interspecific difference in biomass allocation was important in ML and HL, but not in LL where both species were under a high degree of light stress. Consistent with the results of Reich et al. (1998), greater RGR of *P. serotina* resulted from higher LAR and A_L_ compared with *Q. petraea*. The behavior of *P. serotina* in response to increasing light was to some extent similar to that of invasive *Bischofia javanica* Blume which also showed higher morphological plasticity, faster leaf production and higher tolerance to photoinhibition compared with native species (Yamashita et al. 2000, 2002). Interestingly, in our study, the invasive species invested in leaf tissue and high RGR under light deficit and competition/mulching in P + Q + L, but in HL leaf production and RGR were reduced by the competition in P + Q (Fig. 5). These differences between competition treatments were noticed when shoot/root ratios were compared (data not shown). The morphological response of *P. serotina* to interspecific competition was more plastic than that of *Q. petraea*. Our results are in agreement with Kozovits et al. (2005), who showed that *F. sylvatica* displayed smaller crown volumes per unit of shoot biomass in species mixtures compared with monoculture, whereas *P. abies* enhanced space sequestration in mixed culture.

In conclusion, our study shows that light environment has a stronger effect on photosynthesis than the effects of interspecific competition and/or allelopathic effects of *P. serotina* leaves. The response to light of both study species was similar, but in agreement with hypothesis 5, invasive *P. serotina* displayed higher morphological plasticity and was better able to cope with competition than native *Q. petraea*. Our results suggest that the quantum yield of PSII of an invasive species can increase in presence of a competitor and that the higher plasticity of leaf absorbed light energy partitioning appears to give a competitive advantage to invasive *P. serotina* over native *Q. petraea. P. serotina* seedlings allocated proportionally more biomass to aboveground tissues, allowing them to capture a greater amount of light and photosynthesize more efficiently. In contrast, the native seedlings invested more in root development which gave them an advantage in belowground competition for water and nitrogen. Additionally, *Q. petraea* did not decrease area- and mass-based A_max_ and PNUE under the pressure of competition and positively responded to mulching with *P. serotina* leaves. Therefore, despite the similar ecological requirements of invasive *P. serotina* and native *Q. petraea*, they can co-occur in the same environment due to their different strategies of biomass allocation, partitioning of leaf absorbed light energy, and photosynthetic efficiency.

## Electronic supplementary material

Below is the link to the electronic supplementary material.

Supplementary material 1 (PDF 224 KB)

## References

[CR1] Abrams P (1983). The theory of limiting similarity. Annu Rev Ecol Syst.

[CR2] Alba C, Hufbauer R (2012). Exploring the potential for climatic factors, herbivory, and co-occurring vegetation to shape performance in native and introduced populations of *Verbascum thapsus*. Biol Invasions.

[CR500] Bais HP, Vepachedu R, Gilroy S, Callaway RM, Vivanco JM (2003). Allelopathy and exotic plant invasion: from molecules and genes to species interactions. Science.

[CR3] Baker HG, Baker HG, Stebbins GL (1965). Characteristics and modes of origin of weeds. The genetics of colonizing species.

[CR4] Balandier P (2005). Designing forest vegetation management strategies based on the mechanisms and dynamics of crop tree competition by neighbouring vegetation. Forestry.

[CR5] Baruch Z, Goldstein G (1999). Leaf construction cost, nutrient concentration, and net CO_2_ assimilation of native and invasive species in Hawaii. Oecologia.

[CR6] Boyd J, Xu C-Y, Griffin K (2009). Cost-effectiveness of leaf energy and resource investment of invasive *Berberis thunbergii* and co-occurring native shrubs. Can J For Res.

[CR7] Callaway RM, Aschehoug ET (2000). Invasive plants versus their new and old neighbors: a mechanism for exotic invasion. Science.

[CR8] Closset-Kopp D, Chabrerie O, Valentin B, Delachapelle H, Decocq G (2007). When Oskar meets Alice: does a lack of trade-off in r/K-strategies make Prunus serotina a successful invader of European forests?. For Ecol Manag.

[CR9] Closset-Kopp D, Saguez R, Decocq G (2011). Differential growth patterns and fitness may explain contrasted performances of the invasive *Prunus serotina* in its exotic range. Biol Invasions.

[CR10] Craine JM, Dybzinski R (2013). Mechanisms of plant competition for nutrients, water and light. Funct Ecol.

[CR11] Csiszár Á, Korda M, Schmidt D, Šporčić D, Süle P, Teleki B, Tiborcz V, Zagyavai G, Bartha D (2013). Allelopathic potential of some invasive plant species occurring in Hungary. Allelopath J.

[CR12] Daehler CC (2003). Performance comparisons of co-occurring native and alien invasive plants: implications for conservation and restoration. Annu Rev Ecol Sys.

[CR13] Davis MA, Grime JP, Thompson K (2000). Fluctuating resources in plant communities: a general theory of invasibility. J Ecol.

[CR14] Demmig-Adams B, Adams III (1996). Xanthophyll cycle and light stress in nature: uniform response to direct sunlight among higher plant species. Planta.

[CR15] Demmig-Adams B, Adams WW (2006). Photoprotection in an ecological context: the remarkable complexity of thermal energy dissipation. New Phytol.

[CR505] Demmig-Adams B, Adams III WW, Barker DH, Logan BA, Bowling DR, Verhoeven AS (1996). Using chlorophyll fluorescence to assess the fraction of absorbed light allocated to thermal dissipation of excess excitation. Physiol Plantarum.

[CR16] Ellsworth DS, Reich PB (1992). Leaf mass per area, nitrogen content and photosynthetic carbon gain in *Acer saccharum* seedlings in contrasting forest light environmnents. Funct Ecol.

[CR17] Firn J, MacDougall AS, Schmidt S, Buckley YM (2010). Early emergence and resource availability can competitively favour natives over a functionally similar invader. Oecologia.

[CR18] Flexas J, Medrano H (2002). Energy dissipation in C_3_ plants under drought. Funct Plant Biol.

[CR19] Forestry Compendium, Forestry Compendium (2005). Prunus serotina, Quercus petraea. Global edition.

[CR20] Funk JL (2008). Differences in plasticity between invasive and native plants from a low resource environment. J Ecol.

[CR21] Genty B, Briantais J-M, Baker NR (1989). The relationship between the quantum yield of photosynthetic electron transport and quenching of chlorophyll fluorescence. Biochim Biophys Acta.

[CR22] Gleadow RM, Foley WJ, Woodrow IE (1998). Enhanced CO_2_ alters the relationship between photosynthesis and defence in cyanogenic *Eucalyptus cladocalyx* F. Muell. Plant Cell Environ.

[CR23] Grime JP (1974). Vegetation classification by reference to strategies. Nature.

[CR24] Grime JP (1977). Evidence for the existence of three primary strategies in plants and its relevance to ecological and evolutionary theory. Am Nat.

[CR25] Halarewicz A (2011). The reasons underlying the invasion of forest communities by black cherry, *Prunus serotina* and its subsequent consequences. Forest Res Pap.

[CR26] Harrington RA, Brown BJ, Reich PB (1989). Ecophysiology of exotic and native shrubs in Southern Wisconsin. I. Relationship of leaf characteristics, resource availability, and phenology to seasonal patterns of carbon gain. Oecologia.

[CR27] Heberling JM, Fridley JD (2013). Resource-use strategies of native and invasive plants in Eastern North American forests. New Phytol.

[CR28] Hendrickson L, Furbank RT, Chow WS (2004). A simple alternative approach to assessing the fate of absorbed light energy using chlorophyll fluorescence. Photosynth Res.

[CR29] Hierro JL, Callaway RM (2003). Allelopathy and exotic plant invasion. Plant Soil.

[CR30] Hikosaka K, Terashima I (1996). Nitrogen partitioning among photosynthetic components and its consequences in sun and shade plants. Funct Ecol.

[CR506] Hunt R (1982) Plant growth curves. The functional approach to plant growth analysis. Edward Arnold, London

[CR31] Keane RM, Crawley MJ (2002). Exotic plant invasions and the enemy release hypothesis. Trends Ecol Evol.

[CR32] Kenk G (1993). New perspectives in German oak silviculture. Ann For Sci.

[CR33] Koutika L-S, Vanderhoeven S, Chapuis-Lardy L, Dassonville N, Meerts P (2007). Assessment of changes in soil organic matter after invasion by exotic plant species. Biol Fertil Soils.

[CR34] Kozovits AR, Matyssek R, Winkler JB, Göttlein A, Blaschke H, Grams TEE (2005). Above-ground space sequestration determines competitive succes in juvenile beech and spruce trees. New Phytol.

[CR35] Leavesley HB, Li L, Prabhakaran K, Borowitz JL, Isom GE (2008). Interaction of cyanide and nitric oxide with cytochrome c oxidase: implications for acute cyanide toxicity. Toxicol Sci.

[CR36] Lei TT, Lechowicz MJ (1998). Diverse responses of maple saplings to forest light regimes. Ann Bot Lond.

[CR37] Leishman MR, Haslehurst T, Ares A, Baruch Z (2007). Leaf trait relationships of native and invasive plants: community- and global-scale comparisons. New Phytol.

[CR38] Leishman MR, Thomson VP, Cooke J (2010). Native and exotic invasive plants have fundamentally similar carbon capture strategies. J Ecol.

[CR39] Mack RN (1996). Predicting the identity and fate of plant invaders: emergent and emerging approches. Biol Conserv.

[CR40] Mangla S, Sheley RL, James JJ, Radosevich SR (2011). Intra and interspecific competition among invasive and native species during early stages of plant growth. Plant Ecol.

[CR41] Marquis AD, Burns RM, Honkala BH (1990). *Prunus serotina* Ehrh. Black Cherry. Silvics of North America: 2. Hardwoods—Agriculture Handbook 654.

[CR42] Maxwell K, Johnson GN (2000). Chlorophyll fluorescence — a practical guide. J Exp Bot.

[CR43] McDowell SCL (2002). Photosynthetic characteristics of invasive and noninvasive species of *Rubus* (*Rosaceae*). Am J Bot.

[CR44] Molina-Montenegro MA, Penuelas J, Munné-Bosch S, Sardans J (2012). Higher plasticity in ecophysiological traits enhances the performance and invasion success of *Taraxacum officinale* (dandelion) in alpine environments. Biol Invasions.

[CR45] Möllerová J (2005). Notes on invasive and expansive trees and shrubs. J For Sci.

[CR46] Neilson EH, Goodger JQD, Woodrow IE, Møller BL (2013). Plant chemical defense: at what cost?. Trends Plant Sci.

[CR47] Niinemets Ü, Bilger W, Kull O, Tenhunen JD (1998). Acclimation to high irradiance in temperate deciduous trees in the field: changes in xanthophyll cycle pool size and in photosynthetic capacity along a canopy light gradient. Plant Cell Environ.

[CR48] Niyogi KK (1999). Photoprotection revisited: genetic and molecular approaches. Annu Rev Plant Phys.

[CR49] Novoplansky A (2009). Picking battles wisely: plant behaviour under competition. Plant Cell Environ.

[CR50] Oguchi R, Hikosaka K, Hiura T, Hirose T (2008). Costs and benefits of photosynthetic light acclimation by tree seedlings in response to gap formation. Oecologia.

[CR51] Oguchi R, Hiura T, Hikosaka K (2017). The effect of interspecific variation in photosynthetic plasticity on 4-year growth rate and 8-year survival of understorey tree seedlings in response to gap formations in a cool-temperate deciduous forest. Tree Physiol.

[CR52] Parker JD, Torchin ME, Hufbauer RA, Lemoine NP, Alba Ch, Blumenthal DM, Bossdorf O, Byers JE, Dunn AM, Heckman RW (2013). Do invasive species perform better in their new ranges?. Ecology.

[CR53] Pattison RR, Goldstein G, Ares A (1998). Growth, biomass allocation and photosynthesis of invasive and native Hawaiian rainforest species. Oecologia.

[CR54] Patton CA, Ranney TG, Burton JD, Walgenbach JF (1997). Natural pest resistance of *Prunus* taxa to feeding by adult Japanese beetles: Role of endogenous allelochemicals in host plant resistance. J Hortic Sci.

[CR55] Peet R, Christensen N (1987). Competition and tree death. Bioscience.

[CR56] Rascher U, Liebieg M, Lüttge U (2000). Evaluation of instant light-response curves of chlorophyll fluorescence parameters obtained with a portable chlorophyll fluorometer on site in the field. Plant Cell Environ.

[CR57] Reich BP, Walters BM, Ellsworth SD (1992). Leaf life-span in relation to leaf, plant, and stand characteristics among diverse ecosystems. Ecol Monogr.

[CR58] Reich BP, Walters BM, Ellsworth SD, Vose JM, Volin JC, Gresham C, Bowman WD (1998). Relationships of leaf dark respiration to leaf nitrogen, specific leaf area and leaf life-span: a test across biomes and functional groups. Oecologia.

[CR59] Reinhart KO, Packer A, Van der Putten WH, Clay K (2003). Plant-soil biota interactions and spatial distribution of black cherry in its native and invasive ranges. Ecol Lett.

[CR60] Robakowski P (2005). Susceptibility to low-temperature photoinhibition in three conifer tree species differing in successional status. Tree Physiol.

[CR61] Robakowski P, Bielinis E (2011). Competition between sessile oak (*Quercus petraea*) and black cherry (*Padus serotina*): dynamics of seedlings growth. Pol J Ecol.

[CR62] Robakowski P, Bielinis E, Stachowiak J, Mejza I, Bułaj B (2016). Seasonal changes affect root prunasin concentration in *Prunus serotina* and override species interactions between *P. serotina* and *Quercus petraea*. J Chem Ecol.

[CR63] Sendall K, Reich PB (2013). Variation in leaf and twig CO2 flux. as a function of plant size a comparison of seedlings, saplings and trees. Tree Physiol.

[CR64] Swain E, Li CP, Poulton JE (1992). Development of the potential for cyanogenesis in maturing black cherry (*Prunus serotina* Ehrh.) fruits. Plant Physiol.

[CR65] Ubalua AO (2010). Cyanogenic glycosides and the fate of cyanide in soil. Aust J Crop Sci.

[CR66] Valladares F, Wright SJ, Lasso E, Kitajima K, Pearcy RW (2000). Plastic phenotypic response to light of 16 congeneric shrubs from a Panamian rainforest. Ecology.

[CR67] van Kleunen M, Weber E, Fischer M (2010). A meta-analysis of trait differences between invasive and non-invasive plant species. Ecol Lett.

[CR69] Vetter J (2000). Plant cyanogenic glycosides. Toxicon.

[CR70] Wright IJ, Reich PB, Westoby M, Ackerly DD, Baruch Z, Bongers F, Cavender-Bares J, Chapin T, Cornelissen JHC, Diemer M (2004). The worldwide leaf economics spectrum. Nature.

[CR71] Yamashita N, Ishida A, Kushima H, Tanaka N (2000). Acclimation to sudden increase in light favoring an invasive over native trees in subtropical islands. Jpn Oecol.

[CR72] Yamashita N, Koike N, Ishida A (2002). Leaf ontogenetic dependence of light acclimation in invasive and native subtropical trees of different successional status. Plant Cell Environ.

[CR73] Yuan Y, Guo W, Ding W, Wang R (2013). Competitive interaction between the exotic plant *Rhus typhina* L. and the native tree *Quercus acutissima* Carr. in Northern China under different soil N: P ratios. Plant Soil.

[CR74] Zohner CM, Renner SS (2017). Innately shorter vegetation periods in North American species explain native–non-native phenological asymmetries. Nat Ecol Evol.

