# Supplementary material for: Light energy partitioning, photosynthetic efficiency and biomass allocation in invasive Prunus serotina and native Quercus petraea in relation to light environment, competition and allelopathy
Source: J Plant Res. 2018 Feb 7;131(3):505–23. doi: 10.1007/s10265-018-1009-x (PMC5916994; doi:10.1007/s10265-018-1009-x)
Supplement: Supplementary file 1 — Supplementary material 1 (PDF 224 KB) [file 10265_2018_1009_MOESM1_ESM.pdf]

Electronic Supplemental Material

Biomass allocation to foliage overrules photosynthetic responses to competition between invasive *Prunus serotina* and native *Quercus petraea* under different light environments

Piotr Robakowski[*1], Ernest Bielinis[1,2], Kerrie Sendall[3]

[1]Poznan University of Life Sciences, Department of Forestry, Wojska Polskiego 71E St., 60-625 Poznan, Poland; [*]phone: +48 61 848 77 38, fax: +48 61 848 77 34

[2]University of Warmia and Mazury in Olsztyn, Department of Environmental Management and Agriculture, Unit of Forestry and Forest Ecology, PL Lodzki 2, 10-727 Olsztyn, Poland

[3]Georgia Southern University, Department of Biology, P.O. Box 8042, Statesboro, GA 30460, USA

[*]Author for correspondence e-mail: pierrot@up.poznan.pl

Content:

Table S1–S8

Table S1 Analysis of variance in split-plot design of block, light, combination treatments and interactions on quantum yield of constitutive fluorescence and thermal dissipation ($\Phi_{f,D}$), quantum yield of thermal energy dissipation (Φ_{NPQ}), and quantum yield of PSII photochemistry (Φ_{PSII}) of *Prunus serotina* and *Quercus petraea* seedlings at PPF = 295 µmol m^{-2} s^{-1} (P < 0.05; n = 6, n – number of seedlings per species, light and combination treatments).

Effect	*Prunus serotina*			*Quercus petraea*		
	Df	*F*	*P*	*Df*	*F*	*P*
**$\Phi_{f,D}$**						
Block	2	0.75	0.528	2	0.196	0.830
Light	2	34.42	0.003	2	2.784	0.175
Block x light	4	1.12	0.364	4	3.129	0.022
Combination	2	1.56	0.223	3	0.439	0.726
Light x combination	4	1.60	0.195	6	0.604	0.725
**Φ_{NPQ}**						
Block	2	3.20	0.051	2	1.24	0.382
Light	2	5.21	0.010	2	10.26	0.027
Block x light	4	5.34	0.002	4	2.07	0.097
Combination	2	6.19	0.005	3	1.46	0.235
Light x combination	4	1.63	0.187	6	1.55	0.179
**Φ_{PSII}**						
Block	2	5.61	0.007	2	0.88	0.484
Light	2	32.06	0.000	2	6.30	0.058
Block x light	4	6.76	0.000	4	4.10	0.006
Combination	2	6.36	0.004	3	0.865	0.465
Light x combination	4	1.83	0.144	6	1.35	0.254

Table S2 Mean values (±SE) of leaf mass to area ratio (LMA) in *Prunus serotina* and *Quercus petraea* growing in monoculture (P, Q, Q+L) or in competition with *Quercus petraea* and mulching with *P. serotina* leaves (Q+P+L). The same letters indicate that the mean values do not significantly differ in Tukey's test at α < 0.05. Date of sampling – day of year, *n* – number of replications.

Effect		*n*	*P. serotina*			*Q. petraea*			
			Mean		±SE	*n*	Mean		±SE
Date of sampling	177	42	36.89	a	0.94	70	57.86	a	0.98
	207	48	37.54	a	0.78	70	64.09	b	1.06
	238	47	45.26	b	1.17	65	66.83	b	0.92
	269	41	52.66	c	1.67	57	67.47	b	0.99
Light	10%	58	37.65	a	0.33	80	58.26	a	0.72
	25%	62	38.10	a	0.68	91	60.64	b	0.63
	100%	58	53.34	b	1.33	91	72.39	c	0.82
Combination	P	79	43.20		1.32	-	-		-
	Q	-	-		-	87	64.95	ab	1.16
	Q+L	-	-		-	88	66.84	b	1.04
	Q+P+L	99	42.91		1.13	87	61.80	a	0.93
Date of sampling × combination	177 × P	17	36.61		1.67	-	-		-
	177 × Q	-	-		-	24	58.99		2.00
	177 × Q+L	-	-		-	24	58.99		1.77
	177 × Q+P+L	25	37.33		1.57	22	56.86		2.08
	207 × P	22	37.62		1.19	-	-		-
	207 × Q	-	-		-	23	65.00		2.16
	207 × Q+L	-	-		-	22	68.80		2.11
	207 × Q+P+L	26	37.87		1.33	25	61.13		1.95
	238 × P	23	44.21		2.06	-	-		-
	238 × Q	-	-		-	21	68.06		2.43
	238 × Q+L	-	-		-	22	68.45		1.65
	238 × Q+P+L	24	45.96		1.98	22	65.70		1.74
	269×P	17	55.65		3.39	-	-		-
	269 × Q	-	-		-	19	68.99		2.21
	269 × Q+L	-	-		-	20	72.33		1.68
	269 × Q+P+L	24	51.13		2.77	18	63.99		1.99
Light × combination	10% × P	24	40.03		1.45	-	-		-
	10% × Q	-	-		-	26	56.42	ab	1.13
	10% × Q+L	-	-		-	30	63.24	a	1.22
	10% × Q+P+L	34	36.84		0.94	24	55.33	bc	1.38
	25% × P	30	37.44		1.04	-	-		-
	25% × Q	-	-		-	30	61.77	a	1.09
	25% × Q+L	-	-		-	28	63.13	a	1.45
	25% × Q+P+L	32	38.64		1.04	33	58.81	ac	1.04
	100% × P	25	53.17		2.87	-	-		-
	100% × Q	-	-		-	31	75.18	d	1.73
	100% × Q+L	-	-		-	30	73.90	d	1.91
	100% × Q+P+L	33	53.30		2.11	30	70.27	d	1.25
		178				262			

Table S3 Mean values (±SE) of dark respiration (R_d) and maximal net CO_2 assimilation rate (A_{max}) in *Prunus serotina* seedlings growing in monoculture (P) or in competition with *Quercus petraea* with mulching with *P. serotina* leaves (Q+P+L). The same letters indicate that the mean values do not significantly differ in Tukey's test at α < 0.05. Date of sampling – day of year, *n* – number of replications.

Effect		*n*	R_d (nmol g^{-1} s^{-1})			A_{max} (nmol g^{-1} s^{-1})		
			Mean		±SE	Mean		±SE
Date of sampling	177	42	2.80		0.22	27.84		2.38
	207	48	2.83		0.21	26.11		1.93
	238	47	3.30		0.31	32.72		3.20
	269	47	3.84		0.49	28.61		3.79
Light	10%	64	2.32	a	0.17	18.82	a	1.39
	25%	61	2.59	a	0.14	25.52	b	1.01
	100%	59	4.86	b	0.36	43.08	c	3.20
Combination	P	84	3.23		0.26	32.63	a	2.23
	P+Q+L	100	3.17		0.23	25.16	b	1.86
Date of sampling × combination	177 × P	17	2.94		0.38	31.76		3.73
	177 × P+Q+L	25	2.70		0.27	25.07		2.99
	207×P	22	2.56		0.25	25.27		2.51
	207×P+Q+L	26	3.16		0.34	27.13		3.06
	238×P	23	3.17		0.42	37.17		4.23
	238×P+Q+L	24	3.42		0.48	28.28		4.67
	269×P	22	4.30		0.78	36.83		6.05
	269×P+Q+L	25	3.44		0.62	20.87		3.97
Light × combination	10% × P	29	2.32		0.26	22.44		2.14
	10% × P+Q+L	35	2.32		0.24	15.52		1.53
	25% × P	28	2.49		0.24	26.28		1.60
	25% × P+Q+L	33	2.68		0.17	24.83		1.27
	100% ×P	27	4.99		0.52	49.16		4.16
	100% ×P+Q+L	32	4.74		0.51	36.69		4.56
Prunus serotina		184	3.20		0.17	28.80		1.48

Table S4 Mean values (±SE) of dark respiration (R_d) and maximal net CO_2 assimilation rate (A_{max}) in *Quercus petraea* seedlings growing in monoculture (Q) or in competition with *Prunus serotina* with mulching with *P. serotina* leaves (Q+P+L). The same letters indicate that the mean values do not significantly differ in Tukey's test at $\alpha < 0.05$ Date of sampling - day of year, *n* – number of replications.

Effect		*n*	R_d (nmol g^{-1} s^{-1})			A_{max} (nmol g^{-1} s^{-1})		
			Średnia		±SE	Średnia		±SE
Date of sampling	177	70	6.29		0.29	62.41	a	4.11
	207	70	6.00		0.29	49.72	ab	3.83
	238	65	5.90		0.31	43.68	b	3.36
	269	72	6.40		0.31	31.46	c	2.99
Light	10%	85	5.58	a	0.23	28.48	a	1.94
	25%	96	5.61	a	0.20	41.01	b	2.67
	100%	96	7.28	b	0.29	69.38	c	3.13
Combination	Q	91	6.26	ab	0.28	47.30	ab	3.71
	Q+L	94	6.75	a	0.25	50.64	a	3.27
	Q+P+L	82	5.52	b	0.23	42.76	b	3.19
Date of sampling × combination	177×Q	24	6.82		0.51	63.08		7.67
	177× Q+L	24	6.39		0.51	61.02		5.81
	177 × Q+P+L	22	5.67		0.48	63.21		8.27
	207×Q	23	6.20		0.52	51.52		7.65
	207×Q+L	22	6.82		0.52	60.30		7.43
	207×Q+P+L	25	5.24		0.44	40.97		4.87
	238×Q	21	5.76		0.56	41.22		6.67
	238×Q+L	22	6.31		0.46	46.37		5.28
	238×Q+P+L	22	5.67		0.57	43.63		5.68
	269×Q	23	6.23		0.67	33.24		5.94
	269×Q+L	26	7.40		0.47	35.83		5.74
	269×Q+P+L	23	5.54		0.41	25.74		3.78
Light × combination	10% × Q	27	5.36		0.48	22.61	a	2.82
	10% × Q+L	32	6.03		0.40	40.62	bc	3.68
	10% × Q+P+L	26	5.36		0.34	22.24	a	1.80
	25% × Q	31	5.90		0.28	41.59	bc	4.93
	25% × Q+L	31	6.38		0.29	39.50	b	4.73
	25% × Q+P+L	34	4.63		0.34	41.94	bc	4.46
	100% × Q	33	7.43		0.53	72.65	d	5.11
	100% × Q+L	31	7.97		0.49	74.62	d	5.02
	100% × Q+P+L	32	6.57		0.44	62.39	cd	5.71
Quercus petraea		277	6.15		0.15	46.71		1.96

Table S5 Analysis of variance in split-split plot design of block, date of sampling, light and combination treatments and interactions on maximal net CO_2 assimilation rate (A_{crown}, nmol × total seedling foliage dry mass^{-1} × s^{-1}) of *Prunus serotina* and *Quercus petraea* seedlings at $P < 0.05$.

Effect (nmol × g^{-1} × s^{-1})	*Prunus serotina*			*Quercus petraea*		
	df	*F*	*P*	*df*	*F*	*P*
Block	2	0.40	0.400	3	19.89	0.002
Date of sampling	3	171.32	0.000	2	4.29	0.070
Date x block	6	0.41	0.863	6	0.43	0.850
Light	2	83.15	0.000	2	94.66	0.000
Date of sampling x light	6	10.26	0.000	6	1.78	0.168
Block x light + Date x light x block	16	1.15	0.322	16	1.20	0.280
Combination	1	0.46	0.500	2	9.62	0.000
Date x combination	3	1.83	0.149	6	1.01	0.421
Light x combination	2	0.87	0.421	4	2.14	0.080
Date x light x combination	6	0.38	0.892	12	0.62	0.820

Table S6 Mean values (±SE) of maximal net CO_2 assimilation rate (A_{crown}) in *Prunus serotina* and *Quercus petraea* seedlings growing in monoculture (P, Q, respectively) or in competition with mulching with *P. serotina* leaves (Q+P+L). A_{crown} was recalculated per dry mass of total seedling foliage. The same letters indicate that the mean values do not significantly differ in Tukey's test at $\alpha < 0.05$. Date of sampling - day of year, *n* – number of replications.

Effect		*n*	*P. serotina*			*Q. petraea*			
			Mean		±SE	*n*	Mean		±SE
Date of sampling	177	29	2.2	a	0.2	49	29.4	a	2.7
	207	31	8.6	a	1.5	49	23.7	a	2.6
	238	30	54.9	b	11.8	46	34.1	a	4.7
	269	34	70.6	c	21.2	51	21.1	a	3.3
Light	10%	42	3.9	a	0.5	63	10.7	a	1.0
	25%	42	15.6	b	2.3	65	19.7	b	1.5
	100%	40	89.0	c	18.8	67	49.1	c	3.3
Combination	P	60	45.9	a	12.5	-	-		-
	Q	-	-		-	62	25.8	ab	2.4
	Q+L	-	-		-	62	32.1	b	3.4
	Q+P+L	64	25.4	a	6.5	71	23.4	a	2.9
Date of sampling × combination	177 × P	17	36.61		1.67	-	-		-
	177 × Q	-	-		-	24	58.99		2.00
	177 × Q+L	-	-		-	24	58.99		1.77
	177 × Q+P+L	25	37.33		1.57	22	56.86		2.08
	207 × P	22	37.62		1.19	-	-		-
	207 × Q	-	-		-	23	65.00		2.16
	207 × Q+L	-	-		-	22	68.80		2.11
	207 × Q+P+L	26	37.87		1.33	25	61.13		1.95
	238 × P	23	44.21		2.06	-	-		-
	238 × Q	-	-		-	21	68.06		2.43
	238 × Q+L	-	-		-	22	68.45		1.65
	238 × Q+P+L	24	45.96		1.98	22	65.70		1.74
	269×P	17	55.65		3.39	-	-		-
	269 × Q	-	-		-	19	68.99		2.21
	269 × Q+L	-	-		-	20	72.33		1.68
	269 × Q+P+L	24	51.13		2.77	18	63.99		1.99
Light × combination	10% × P	24	40.03		1.45	-	-		-
	10% × Q	-	-		-	26	56.42	ab	1.13
	10% × Q+L	-	-		-	30	63.24	a	1.22
	10% × Q+P+L	34	36.84		0.94	24	55.33	bc	1.38
	25% × P	30	37.44		1.04	-	-		-
	25% × Q	-	-		-	30	61.77	a	1.09
	25% × Q+L	-	-		-	28	63.13	a	1.45
	25% × Q+P+L	32	38.64		1.04	33	58.81	ac	1.04
	100% × P	25	53.17		2.87	-	-		-
	100% × Q	-	-		-	31	75.18	d	1.73
	100% × Q+L	-	-		-	30	73.90	d	1.91
	100% × Q+P+L	33	53.30		2.11	30	70.27	d	1.25
		178				262			

Table S7 Mean values (±SE) of photosynthetic nitrogen use efficiency (PNUE) and water use efficiency for photosynthesis (WUE) in *Prunus serotina* seedlings growing in monoculture (P) or in competition with *Quercus petraea* with mulching with *P. serotina* leaves (Q+P+L). The same letters indicate that the mean values do not significantly differ in Tukey's test at $\alpha < 0.05$ Date of sampling - day of year, *n* – number of replications.

Effect		*n*	PNUE (μmol CO_2 mol N^{-1} s^{-1})		*n*	WUE (μmol CO_2 mmol H_2O^{-1})	
			Mean	±SE		Mean	±SE
Date of sampling	177	42	84.13 a	7.40	42	6.86 a	0.23
	207	-	-	-	48	7.09 a	0.23
	238	47	69.35 ab	5.40	47	8.11 b	0.16
	269	47	45.39 b	3.67	47	9.17 b	0.27
Light	10%	48	43.84 a	4.28	64	7.60 a	0.20
	25%	44	77.36 b	5.38	61	7.37 a	0.26
	100%	44	67.35 b	6.20	59	8.63 b	0.24
Combination	P	62	71.23 a	4.79	84	7.80	0.22
	P+Q+L	74	54.36 b	4.57	100	7.88	0.19
Date of sampling × combination	177 × P	17	93.74	10.79	17	7.04	0.36
	177×P+Q+L	25	74.53	9.65	25	6.74	0.30
	207 × P	-	-	-	22	6.90	0.35
	207 ×P+Q+L	-	-	-	26	7.32	0.29
	238 ×P	23	80.42	6.31	23	7.93	0.18
	238 ×P+Q+L	24	57.49	7.95	24	8.27	0.27
	269 ×P	22	49.95	4.99	22	9.21	0.46
	269 ×P+Q+L	25	41.10	5.30	25	9.14	0.31
Light × combination	10% × P	22	48.48	5.54	29	7.64	0.26
	10% × P+Q+L	26	39.53	6.43	35	7.56	0.30
	25% × P	19	84.77	7.87	28	7.12	0.35
	25% × P+Q+L	25	70.47	7.14	33	7.59	0.37
	100% × P	21	79.77	7.85	27	8.69	0.42
	100% × P+Q+L	23	52.85	8.29	32	8.57	0.24
Prunus serotina		136	62.79	3.42	184	7.84	0.14

Table S8 Mean values (±SE) of photosynthetic nitrogen use efficiency (PNUE) and water use efficiency for photosynthesis (WUE) in *Quercus petraea* seedlings growing in monoculture (Q) or in competition with *Prunus serotina* with mulching with *P. serotina* leaves (Q+P+L). The same letters indicate that the mean values do not significantly differ in Tukey's test at $\alpha < 0.05$. Date of sampling - day of year, *n* – number of replications.

Effect		*n*	PNUE (µmol CO_2 mol N^{-1} s^{-1})			*n*	WUE (µmol CO_2 mmol H_2O^{-1})		
			Mean		±SE		Mean		±SE
Date of sampling	177	70	74.77	a	3.80	70	8.37	a	0.26
	207	-	-	-	-	70	7.29	b	0.26
	238	65	47.70	b	3.51	65	6.76	b	0.26
	269	72	32.67	c	2.42	72	6.13	c	0.39
Light	10%	62	36.72	a	2.58	85	6.03	a	0.23
	25%	73	49.27	b	4.59	96	6.75	b	0.24
	100%	72	67.20	c	3.80	96	8.53	c	0.27
Combination	Q	68	49.72		3.90	91	6.94	a	0.29
	Q+L	72	51.52		3.60	94	7.63	b	0.29
	Q+P+L	67	52.79		4.90	92	6.87	a	0.25
Date of sampling × combination	177 × Q	24	72.05		5.75	24	8.26		0.51
	177 × Q+L	24	70.52		4.86	24	8.60		0.33
	177 × Q+P+L	22	82.73		9.09	22	8.23		0.52
	207 × Q	-	-	-	-	23	6.72		0.48
	207 ×Q+L	-	-	-	-	22	8.14		0.32
	207 ×Q+P+L	-	-	-	-	25	7.13		0.47
	238 ×Q	21	45.40		6.26	21	6.21		0.51
	238 ×Q+L	22	47.79		6.34	22	7.10		0.44
	238 ×Q+P+L	22	49.78		6.00	22	6.95		0.39
	269 ×Q	23	31.45		3.64	23	6.45		0.71
	269 ×Q+L	26	36.71		4.45	26	6.69		0.86
	269 ×Q+P+L	23	29.59		4.44	23	5.31		0.42
Light × combination	10% × Q	20	32.18		4.55	27	5.29		0.37
	10% × Q+L	24	44.68		5.14	32	6.93		0.43
	10% × Q+P+L	18	33.03		3.02	26	5.79		0.30
	25% × Q	23	49.67		7.68	31	6.58		0.39
	25% × Q+L	24	40.61		6.30	31	6.84		0.44
	25% × Q+P+L	26	57.55		9.44	34	6.81		0.44
	100% × Q	25	64.39		5.54	33	8.54		0.46
	100% × Q+L	24	68.59		5.08	31	9.33		0.47
	100% × Q+P+L	23	69.09		9.31	32	7.92		0.45
Quercus petraea		207	51.33		2.39	277	7.13		0.16
